# Palladium and Copper: Advantageous Nanocatalysts for Multi-Step Transformations

**DOI:** 10.3390/nano11081891

**Published:** 2021-07-23

**Authors:** Antonio Reina, Trung Dang-Bao, Itzel Guerrero-Ríos, Montserrat Gómez

**Affiliations:** 1 Departamento de Química Inorgánica y Nuclear, Facultad de Química, Universidad Nacional Autónoma de México, Ciudad de México 04510, Mexico; itzelgr@unam.mx; 2 Faculty of Chemical Engineering, Ho Chi Minh City University of Technology (HCMUT), 268 Ly Thuong Kiet Street, District 10, Ho Chi Minh City 700000, Vietnam; 3 Vietnam National University—Ho Chi Minh City (VNU—HCM), Ho Chi Minh City 700000, Vietnam; 4 Laboratoire Hétérochimie Fondamentale et Appliquée, Université Toulouse 3—Paul Sabatier, UMR CNRS 5069, 118 Route de Narbonne, CEDEX 9, 31062 Toulouse, France; gomez@chimie.ups-tlse.fr

**Keywords:** palladium, copper, nanoparticles, catalysis, multi-step transformations

## Abstract

Metal nanoparticles have been deeply studied in the last few decades due to their attractive physical and chemical properties, finding a wide range of applications in several fields. Among them, well-defined nano-structures can combine the main advantages of heterogeneous and homogeneous catalysts. Especially, catalyzed multi-step processes for the production of added-value chemicals represent straightforward synthetic methodologies, including tandem and sequential reactions that avoid the purification of intermediate compounds. In particular, palladium- and copper-based nanocatalysts are often applied, becoming a current strategy in the sustainable synthesis of fine chemicals. The rational tailoring of nanosized materials involving both those immobilized on solid supports and liquid phases and their applications in organic synthesis are herein reviewed.

## 1. Introduction

From a chemical and economic viewpoint, environmentally friendly protocols, in particular multi-step reactions, stand for straightforward synthetic methodologies to obtain added-value chemicals, avoiding intermediate purifications and reducing wastes [1,2,3]. Transition-metal-based catalysts have been applied in such processes and are categorized in two main approaches: (i) multi-step transformations catalyzed by monometallic catalytic systems, which generally exhibit a limited scope; and (ii) multi-step transformations catalyzed by multi-metallic catalysts taking advantage of the properties of each metal and plausible synergies between them. Although the latter seems to be more adapted, the incompatibility between metals often prevents their applications in organic syntheses [1,2,3]. In most cases, homogeneous organometallic complexes emerge as robust and efficient catalysts, regardless of their sensitivity and difficulty in handling and operation. Furthermore, their difficult separation from organic products and poor recyclability result in increased costs and the generation of waste. The contamination of organic phases with metals, even after multiple purifications, raise challenges in the large-scale synthesis of natural compounds in the pharmaceutical industry. Clearly, multi-step processes aim to reduce the operation steps, as well as the economic cost and environmental impact.

The late 1990s witnessed the development of nanoscience and nanotechnology, leading to the design of well-defined metal nano-structures along with nanocatalysis. The field of nanocatalysis has offered a revolutionary change by combining the advantages of both classical catalytic systems (heterogeneous and homogeneous systems), leading to high efficiency and selectivity, and even envisaging new reactivity [4,5,6]. More recently, metal-based nanoparticles have enabled multi-step transformations by the rational tailoring of nanoparticle-based materials (size, shape, structure), including those immobilized on solids and liquids [4,5]. Without a doubt, organic syntheses catalyzed by metallic nanoparticle-based systems usually display lower reactivity than those of homogeneous catalysts. Nonetheless, some outstanding advantages are offered in terms of (i) feasibility of catalysts separation; (ii) preclusion of metal contamination of organic phase; (iii) recyclable ability of expensive metals and (iv) minimizing or avoiding the use of ligands as stabilizers of MNPs without affecting the catalyst performance.

Regarding the catalytic use of metallic nanoparticles, in particular those of palladium and copper, as a current strategy in organic synthesis in virtue of their wide scope of chemical reactions compared to homogeneous catalytic systems, in this review, the state of the art on the preparation of fine chemicals from multi-step transformations from a sustainable point of view is analyzed [7,8,9,10,11,12]. During the last decade, a prominent review was reported by Buchwald et al. (2016) regarding homogeneous palladium-catalyzed multi-step transformations, mainly in C–N cross-coupling reactions [7]. Notwithstanding, there is room for comprehensive reviews focusing on tandem and sequential processes catalyzed by palladium nanoparticles (PdNPs) and copper nanoparticles (CuNPs), including catalyst preparation and the corresponding characterization.

Herein, we analyze the works reported in the literature concerning monometallic PdNPs and CuNPs and well-defined bimetallic nanoparticles (PdCu-BMNPs), which involve the synthesis and characterization of the nanocatalysts applied in multi-step processes, highlighting rational and mechanistic approaches.

## 2. Palladium Nanoparticles

### 2.1. PdNPs Immobilized on Solids

#### 2.1.1. Multi-Step Condensation/Reduction Reactions

**Scheme 1:**
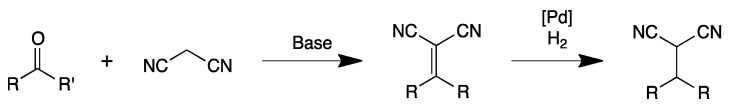
General sequential Knoevenagel condensation/reduction reaction.

Knoevenagel condensation is a valuable approach for the synthesis of α,β-unsaturated carbonyl compounds exhibiting interesting applications as drugs, dyes, pigments and agrochemicals [13]. One-pot synthesis of α-alkylated nitriles by sequential Knoevenagel condensation and reduction of the carbon–carbon double bond is an attractive methodology in terms of sustainable processes to obtain high-value-added products (Scheme 1) [14,15]. Hence, benzylmalonitrile derivatives are often used as key intermediates in a large variety of organic transformations [16]. In general, this reaction is conducted via a sequential methodology consisting of two steps; first, a condensation reaction catalyzed by a Brønsted base to produce an α-alkenyl nitrile which is reduced in the second step by a transition metal-catalyzed hydrogenation [14].

To overcome both the use of two different catalytic systems under different conditions and purification of intermediates, an elegant approach is to apply bifunctional catalytic materials bearing both active sites, which permit to carry out the reaction under one-pot tandem conditions. Functionalized silica, zeolites and metal–organic frameworks (MOF) are among the most recurrent supports used in the design of these bifunctional catalysts [17,18].

**Figure 1:**
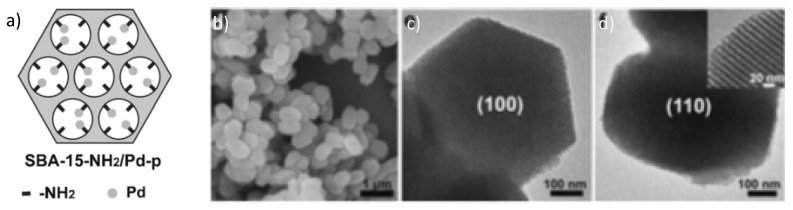
PdNPs anchored to amino-functionalized mesoporous silica (**a**), SEM image of SBA-15-NH_2_/Pd–p (**b**), and TEM images of the hexagonal platelet morphology ((**c**,**d**)). Inset in (**d**) shows high-magnification image [20]. Reproduced from [20] with permission from Wiley.

In 2005, Kaneda and co-workers reported the synthesis of α-alkylated nitriles using hydrotalcite-supported PdNPs [19]. Hydrotalcites, of general formula Mg_6_Al_2_CO_3_(OH)_16_·4H_2_O, exhibit good adsorption characteristics and a basic surface which made them promising materials for this reaction. Transmission Electron Microscopy (TEM) showed the formation of spherical and small PdNPs (ca. 7 nm), showing good control during synthesis. The as-prepared catalytic material was employed under smooth conditions (1 mol% Pd, entry 1, Table 1) for a variety of substrates, including aryl, heteroaryl and alkyl aldehydes and cyclohexyl ketone. The catalytic system showed better performance than PdNPs supported on monometallic oxide supports such as Al_2_O_3_, MgO, Al(OH)_3_ or Mg(OH)_2_. To conclude on a synergic effect of the bifunctional catalyst, a mixture of oxides and carbonates should be used. Song et al. carried out the condensation/hydrogenation reaction by using a bifunctional material constituted of PdNPs anchored to aminopropyl-functionalized mesoporous silica (Figure 1) [20]. The hexagonal platelet morphology of mesoporous silica improved the catalytic activity compared to that exhibiting a fiber morphology due to its shorter length, lower pore congestion and higher mass transportation. PdNPs were not observed by TEM owing to high and uniform dispersion of very small metal nanoparticles, as claimed by the authors; by X-ray photoelectron spectroscopy (XPS), only Pd(0) was detected. In the catalyzed condensation/hydrogenation reaction, high yields at short times (20 min for condensation and 30 min for hydrogenation; entry 2, Table 1) were obtained for a variety of products, including aliphatic aldehydes.

Huang et al. synthesized PdNPs supported on bipyridyl aluminum-based MOF [21]. The Bipyridyl group acts as a Lewis base and also facilitates the stabilization of PdNPs on the support. Very small PdNPs (ca. 1 nm) highly dispersed on the *N*-doped MOF were obtained and efficiently used for the Knoevenagel condensation–hydrogenation sequential reaction. This one-pot process (involving two steps without separation of intermediate products) led to the desired products with high selectivity under relatively harsh conditions (entry 3, Table 1). The same group developed a catalyst based on PdNPs supported on amino-functionalized porous carbon nitride nanosheets [22]. Porous carbon nitride was exposed to ammonia in order to exfoliate the bulk material and enrich the surface in nitrogen atoms (C_3_N_4_-NH_2_). H_2_[PdCl_4_] was reduced under hydrogen to give small crystalline nanoparticles (3.3–3.5 nm) and further supported on the modified carbon nitride. This catalyst afforded α-alkylated nitriles under smooth conditions (1 mol% Pd, entry 4, Table 1). The reaction was enlarged to the use of cyclic, alkyl, aryl and alkyl–aryl ketones as substrates obtaining good to excellent conversion and selectivity. In addition, the catalyst was recycled five times, showing slightly bigger particles after the fifth run.

**Figure 2:**
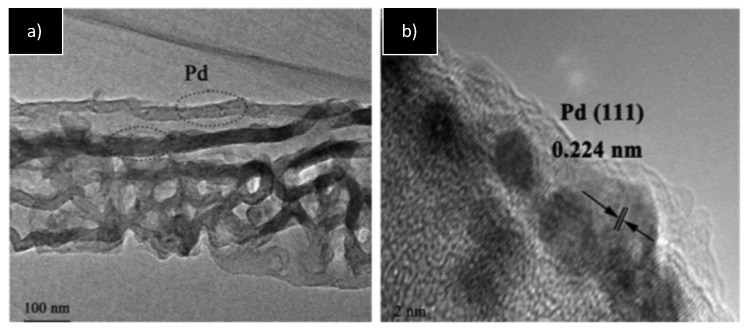
TEM images of the PdNPs on triazine-based-g-C_3_N_4_ nanotubes (**a**,**b**). Inset in (**b**) shows crystallographic planes of *fcc* Pd(0) [24]. Reproduced from [24] with permission from Elsevier.

Wang’s group studied the same type of reaction using anchored PdNPs on a zeolitic imidazolate framework (ZIF) and supported on graphene oxide [23]; palladium precursor was first reduced by NaBH_4_ using PVP (polyvinylpyrrolidone) as a stabilizer, and the PdNPs formed were then anchored on ZIF and supported on graphene oxide. This material showed the presence of crystalline face-centered cubic (fcc) Pd(0) dispersed on the support by XRD. The small palladium-based nanoparticles (ca. 2.8 nm) were constituted by a mixture of Pd(0)/Pd(II) as revealed by XPS. This system was active for the catalyzed Knoevenagel/hydrogenation reaction under relatively harsh conditions (9 mol% Pd), giving the desired products with high selectivity (entry 5, Table 1). The catalyst was recycled up to eight times without loss of activity. Later, the same group improved the catalytic system by using triazine-based carbon nitride nanotubes as support instead of ZIF obtaining slightly bigger Pd(0) nanoparticles (ca. 3.8 nm) (Figure 2) [24]. The high basicity of the support, measured by CO_2_-temperature-programmed desorption (CO_2_-TPD), enabled the catalytic reaction to work under smoother conditions (1 mol% Pd, entry 6, Table 1), obtaining similar conversion and selectivity than that observed with ZIF on graphene oxide. The catalyst was recycled five times without signs of deactivation.

**Scheme 2:**
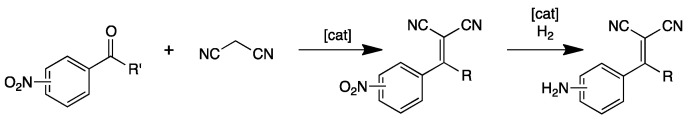
General sequential Knoevenagel condensation followed by selective nitroarene hydrogenation.

In addition to α-alkylated nitriles, sequential Knoevenagel/reduction reaction can be applied to the preparation of 2-(4-aminobenzylidene)-malonitrile, a key intermediate in the synthesis of antihypertensive drugs [25]. In order to obtain this product, after condensation, a selective reduction of nitroarene over carbon–carbon double bond is required (Scheme 2).

**Figure 3:**
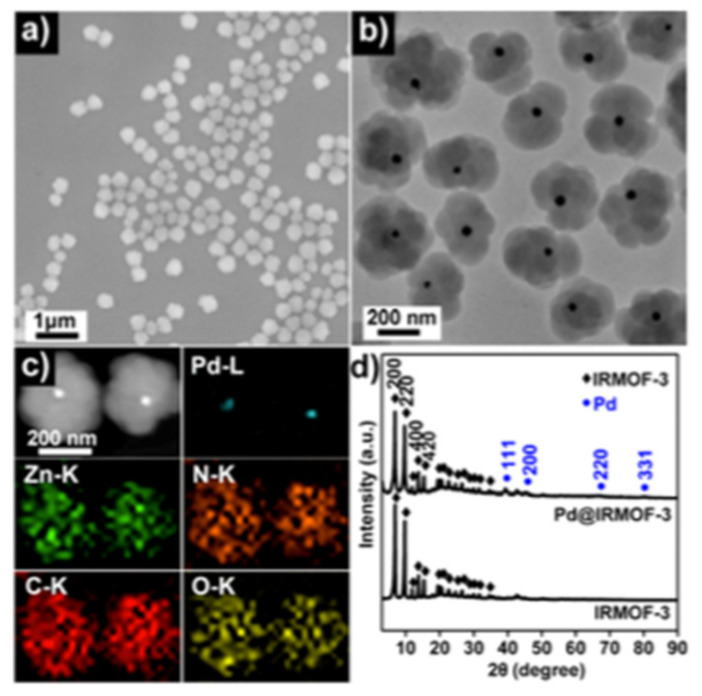
SEM image of the core–shell PdNPs@IRMOF-3 material (**a**), TEM image of the material (**b**), HAADF-STEM image with the corresponding EDX elemental mapping (**c**), PXRD of PdNPs@IRMOF-3 and pure IRMOF-3 (**d**) [27]. Reproduced from [27] with permission from the American Chemical Society.

Chemoselective reduction of nitro compounds is an important area for industrial catalysis because the resulting amines are raw materials for pharmaceutical or agrochemical products [26] Tang et al. addressed this issue using a bifunctional catalyst consisting of core–shell PdNPs-amino-functionalized MOF [27]. Large PdNPs (ca. 35 nm) stabilized by PVP constitute the core and Zn-based amino-functionalized MOF (IRMOF-3), the shell (ca. 145 nm), as evidenced by high-angle annular dark-field (HAADF) imaging coupled to Energy Dispersive X-ray (EDX) elemental mapping. Powder X-ray diffraction (PXRD) showed the *fcc* structure of Pd(0) and the cubic structure of the shell (Figure 3). Interestingly, EtOH/DMF solvent mixture was crucial to tune nucleation and the growth of shell on PdNPs. The as-prepared material was employed in sequential condensation/nitro reduction catalysis, exhibiting good performance (86% selectivity, TOF 3.6 h^−1^) under mild conditions (room temperature, 1 mol% Pd, 2 bar H_2_). Authors experimentally proved that supporting PdNPs on the same modified MOF instead of preparing a core–shell structure resulted in selectivity loss. To explain this behavior, DFT calculations revealed that long-chain molecules are compelled to enter in one single orientation into the MOF well-defined pore. The amino groups at the surface of the shell preferentially interact with the NO_2_ group of the substrate rather than with the C=O group.

Tao et al. prepared a magnetic multicomponent catalyst for the Knoevenagel condensation and selective reduction of nitroarene [28]. Iron oxides and PdNPs were alternately loaded on hierarchically mesoporous silica using a bio-based, polyphenol-assisted layer-by-layer coating. This strategy improved the dispersion of metal nanoparticles on the surface, reduced the blocking of pores and helped the reduction of metal precursors. Various reactions were successfully achieved using this magnetically separable catalyst, including Suzuki–Miyaura coupling and Friedel–Crafts alkylation. Concerning the sequential condensation/hydrogenation reaction, one example gave quantitative yield under harsher conditions than the ones used by Tang (80 °C instead of room temperature). In this case, the catalyst was recycled more than 10 times without activity loss. Dong’s group reported PdNPs-loaded and polymer-grafted on MOF-based catalyst [29]. The pH-sensitive polymer enables the material to conduct the reaction in stable toluene-in-water emulsion by tuning the pH. Emulsification, at neutral conditions, and demulsification, at acidic conditions, control and permit to separate and recycle the catalyst. As a result, aminobenzylidene nitriles with high conversions and selectivity under mild conditions were obtained (room temperature, 30 min). Recently, Anh and co-workers reported a highly active and selective catalyst based on PdNPs supported on amine-functionalized aromatic porous polymer (DETA-APP) and using formic acid as a hydrogen source [30], TEM analyses exhibited the formation of small, spherical and crystalline well-dispersed PdNPs (1–3 nm). Anchoring occurred through electrostatic interaction between Pd and NH_2_-group, as shown by the appearance of new binding energy in the N 1s region of the XPS spectra. One-pot sequential methodology, in which HCOOH was added after 1 hour of reaction, led to the desired products in a relatively high reaction rate (TOF 18.7 h^−1^) with high selectivity (95–99%). PdNPs/DETA-APP is so far the most active catalyst reported in the literature for the sequential Knoevenagel condensation/selective nitro reduction reaction (Table 2, entry 4).

**Scheme 3:**
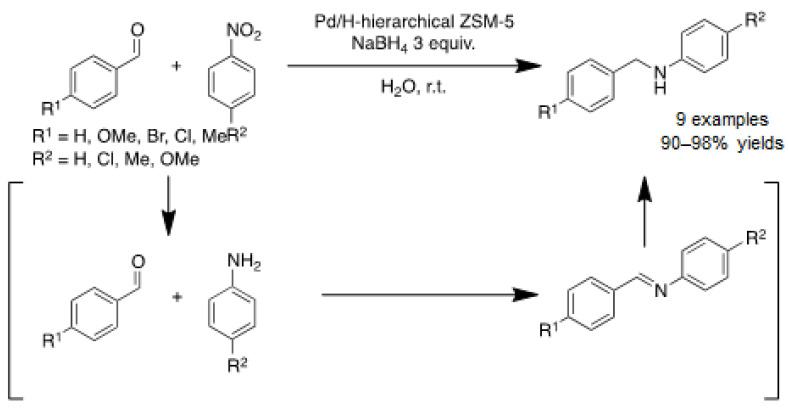
Reductive amination of aldehydes from nitroarenes catalyzed by Pd/H-hierarchical ZSM-5 [31].

Direct reductive amination of aldehydes and ketones with amines is an efficient synthetic approach to perform monoalkylation of amines. This involves a two-step reaction consisting of the condensation of the amine with the corresponding aldehyde to form an imine intermediate, which is then reduced to form a secondary amine. Mazaheri and co-workers also prepared secondary amines through tandem condensation/reduction reaction from nitrobenzene and aldehydes [31]. In this case, nitrobenzene is first reduced to aniline, which condenses with the corresponding aldehyde to form an imine, and the imine reduction yields the desired product. For that, a bifunctional catalyst based on PdNPs supported on acidic hierarchical zeolite (ZSM-5) was developed. Full characterization of the material showed that small spherical PdNPs (ca. 3–6 nm by TEM) were well-dispersed in the mesopores of the zeolite crystalline cubic structure. Reduction of the metal precursor with hydrazine gave only Pd(0), as shown by UV-vis and XPS spectroscopy. The catalytic reaction was conducted in water through a tandem methodology, leading to the formation of benzyl–phenyl secondary amines, for the most part bearing electron-donor groups (Scheme 3). Unfortunately, the catalyst recycling was not efficient due to metal leaching.

**Scheme 4:**
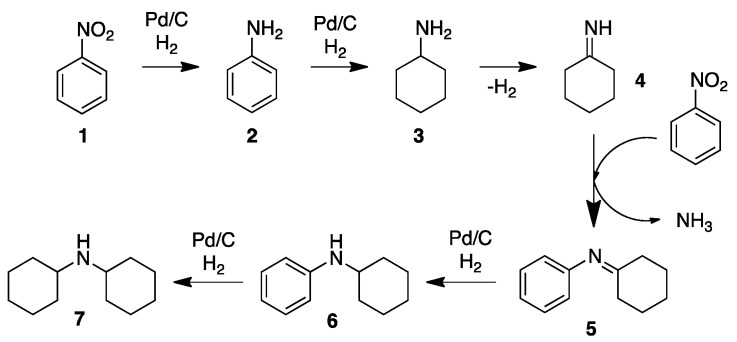
Proposed mechanism for the Pd/C catalyzed hydrogenation of nitrobenzene (**1**) to dicyclohexylamine (**7**) [32].

A different approach for the synthesis of secondary aryl–alkyl or alkyl–alkyl amines was reported by Corma and co-workers [32]. Using commercially available PdNPs on carbon (2.3 nm face-centered cubic Pd(0) particles) and nitroarenes, a variety of aryl–alkyl secondary amines were obtained working at room temperature; at higher temperature (60 °C), alkyl–alkyl secondary amines were also efficiently prepared. In order to achieve this, nitrobenzene was first hydrogenated into aniline **2** (Scheme 4). Further hydrogenation of the aromatic ring gave cyclohexylamine **3**, which triggered a dehydrogenation, affording cyclohexylideneamine **4**. The resulting imine underwent a condensation with nitrobenzene, obtaining ammonia as a concomitant product. Imine **5** was hydrogenated to give cyclohexylaniline, **6**. Further hydrogenation of 6 enabled the formation of dicyclohexylamine **7**. The catalyst was recycled up to 4 times at the gram scale. The same methodology was applied for the synthesis of 1-indoline, well-known for its biological activity, via intramolecular reaction from β,2-dinitrostyrene [33,34].

**Scheme 5:**
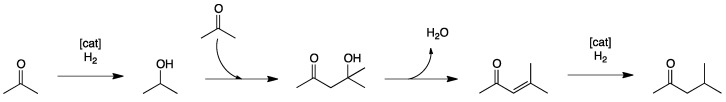
Synthesis of MIBK from multi-step condensation/reduction reaction [35].

Another industrially important multi-step condensation/reduction reaction involves aldol condensation for the synthesis of functionalized ketones. In particular, methyl isobutyl ketone (MIBK) is the most important product derived from acetone, mainly used as a solvent for cellulose-based coating systems and as an initiator for polymerization processes. MIBK is prepared as shown below (Scheme 5). One of the important challenges of this transformation is to selectively obtain MIBK avoiding the formation of several possible side-products (propene, isobutene, isophorone, etc.).

**Scheme 6:**
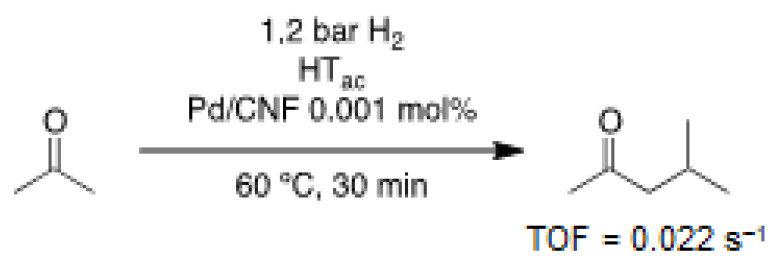
Tandem process catalyzed by a mixture of hydrotalcite and PdNPs on CNF for the synthesis of MIBK [38].

Yang and Wu used PdNPs supported on aluminophosphate molecular sieves for the one-step continuous flow synthesis of MIBK [35], evidencing the importance of the support basicity to improve the selectivity of the reaction. Das and co-workers prepared PdNPs supported on Mg and Al mixed oxides, Pd/MgAl(O) [36,37], obtaining MIBK in good selectivity (75%) at low Pd loading (0.2 wt%). Later, de Jong and co-workers developed a tandem catalytic process for the synthesis of MIBK [38], using a dual catalytic system consisting of PdNPs supported on carbon nanofibers (CNF) and highly basic Mg/Al hydrotalcite (HT_ac_) (Scheme 6).

**Scheme 7:**
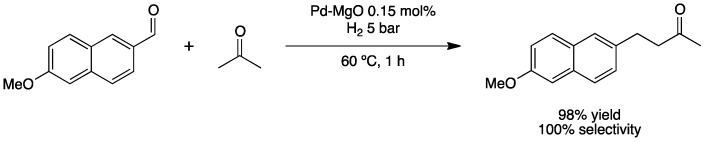
Synthesis of Nabumetone via tandem aldolization/crotonization/hydrogenation reaction catalyzed by PdNPs on MgO (Pd–MgO) [39].

Besides MIBK, Pd-catalyzed multi-step aldolization–crotonization–hydrogenation process (Scheme 7) can be applied in the synthesis of Nabumetone, an anti-inflammatory and analgesic drug applied in rheumatic and arthritic diseases. Corma’s group reported a one-pot direct synthesis of Nabumetone under relatively mild conditions (60 °C, 1 h, 5 bar of H_2_) [39]. PdNPs supported on MgO catalyzed the aldolization/crotonization/hydrogenation tandem reaction, providing excellent yield. MgO exhibits a surface structure characterized by a significant number of defects, where the basic oxide sites placed at faces, edges and corners show low coordination numbers, playing a crucial role in the aldolization/crotonization steps. Recycling of the catalytic system was possible prior to a calcination regeneration step.

**Scheme 8:**
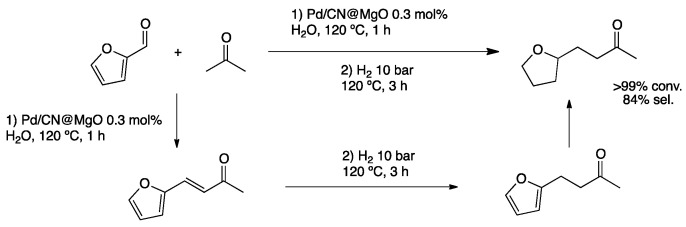
Sequential aldolization/crotonization/hydrogenation reaction of furfural and acetone in water catalyzed by PdNPs stabilized by *N*-doped carbon (CN) supported on MgO [40].

Furfural, a biomass-derived substrate easily accessible from saccharides, can be transformed into high-value-added molecules through sequential condensation–hydrogenation reactions (Scheme 8). Interestingly, 1-(α-tetrahydrofuryl)butan-3-one, which offers various options for further functionalization, can be obtained from furfural and acetone. Wang et al. prepared MgO nanoparticles functionalized with *N*-containing carbon (CN) coated with PdNPs [40]. Full characterization of the hybrid material showed the formation of well-dispersed small PdNPs (ca. 2.2 nm) in the absence of any added stabilizer. The sequential reaction in water within short times (90 min) afforded the desired product in high yield. The catalytic material was recycled four times.

#### 2.1.2. Multi-Step Oxidation Reactions

**Scheme 9:**
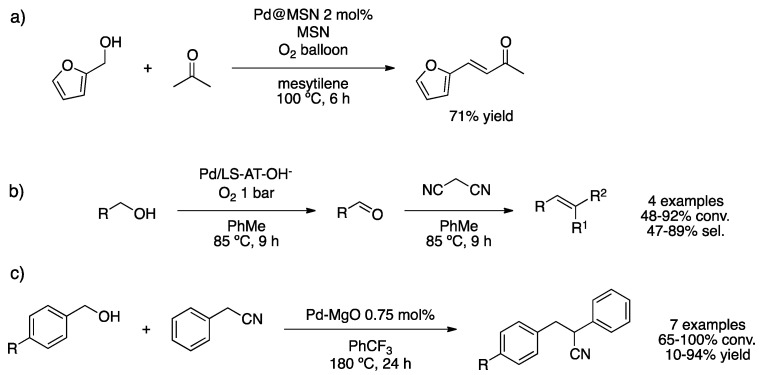
Tandem oxidation/aldol condensation catalyzed by a mixture of PdNPs supported on amino-functionalized MSN (**a**) [41]. Aerobic oxidation/Knoevenagel condensation sequential reaction catalyzed by PdNPs supported on zeolite multilamellar nanosheets (Ls-AT-OH^-^) (**b**) [42]. Tandem oxidation/condensation/reduction catalyzed by PdNPs supported on MgO (**c**) [44].

Selective oxidation of alcohols in a liquid phase is of great importance for industrial purposes. Conventional methodologies include the use of stoichiometric amounts of metal oxidants, such as chromate and permanganate salts, which lead to the generation of toxic wastes. Preferably, metal-catalyzed oxidation using molecular oxygen appears as an elegant low-cost, environmentally friendly alternative. However, the use of molecular oxygen represents major safety problems, and air should be aimed instead. Multi-step oxidation/condensation reactions permit a facile strategy to obtain value-added compounds. Slowing and co-workers reported a dual catalytic system composed of small PdNPs (ca. 2.7 nm) supported on mesoporous silica nanoparticles (MSN) and amino-functionalized MSN for the tandem oxidation/aldol condensation reaction of furfuryl alcohol and acetone (Scheme 9a) [41]. Despite the good yield obtained under low pressure of O_2_, the catalyst was directly recycled due to its deactivation by oxidation, and a regeneration treatment with NaBH_4_ was necessary. Wu et al. studied the sequential aerobic oxidation/Knoevenagel condensation reaction to obtain benzylidenemalonitrile, an intermediate in the synthesis of drugs [42]. The controlled synthesis of PdNPs on multilamellar silicalite zeolite was successfully achieved by using organic structure-directing agents (OSDA) as surfactants. Excess of surfactant was removed by acidic treatment, and then the material was treated with aqueous ammonia to recover its basic properties. XPS analysis exhibited only Pd(0), and TEM images evidenced the high dispersion of small PdNPs on zeolite multilamellar nanosheets (Ls-AT-OH^-^). Sequential reaction afforded the desired products in moderate to good yields without the addition of an external base (Scheme 9b). Li and co-workers employed PdNPs supported on mesoporous carbon nitride for a similar reaction under a continuous flow of molecular oxygen, an appropriate methodology in particular from a safety point of view [43]. XPS spectroscopy revealed that reduction of PdCl_2_ with NaBH_4_ was not efficient, only 31% Pd(0); furthermore, TEM exhibited a broad size distribution of PdNPs. This material gave poor results in the synthesis of methyl benzylidenecyanoacetate. However, this as-prepared material was actually very active for the oxidation of benzyl alcohol into benzaldehyde, and the catalyst was recycled five times. Corma’s group used PdNPs supported MgO as a catalyst for the one-pot tandem oxidation/Knoevenagel condensation/reduction reaction without molecular oxygen nor hydrogen [44]. Oxidation of benzyl alcohol derivative at the surface of the material provided the corresponding aldehyde that underwent Knoevenagel condensation in the presence of benzyl cyanide; the resulting carbon–carbon double bond was reduced by palladium hydride to obtain the desired product (Scheme 9c). Kinetic studies showed that the transfer hydrogenation by the Pd–hydride species is the rate-determining step.

**Scheme 10:**
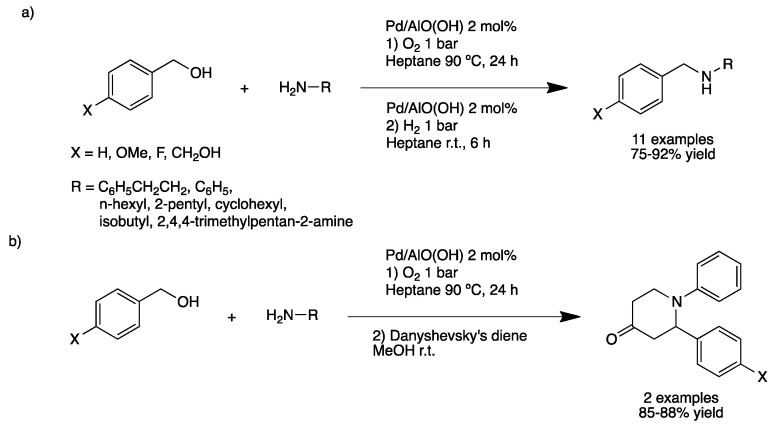
One-pot synthesis of secondary amines from benzyl alcohols: sequential oxidative/reductive process (**a**); sequential oxidative/Aza–Diels–Alder process (**b**) [52].

A straightforward strategy to prepare secondary amines is the *N*-monoalkylation of alcohols with amines. Following the work of Watanabe and Grigg, using Ru, Rh and Ir catalysts for the *N*-alkylation of amines with alcohols, other catalytic systems have been developed including silver nanoparticles supported on alumina and nanosized zeolites [45,46,47,48,49,50]. Concerning supported palladium, Park et al. synthetized aluminum hydroxide-supported PdNPs (Pd/AlO(OH), 2–3 nm as shown by high-resolution TEM) [51]; this catalytic material showed high activity for *N*-alkylation of amines with alcohols [52]. One-pot synthesis of secondary amines by a sequential oxidative/reductive methodology gave high isolated yields of the desired monoalkylated amine from a variety of amines, including aryl and alkyl amines but limited to benzyl alcohol derivatives (Scheme 10a). In addition, the same catalytic system under slightly different conditions was used for the one-pot oxidative reaction followed by an Aza–Diels–Alder reaction to yield biologically valuable, nitrogen-containing six-membered rings in excellent yields (Scheme 10b) [53].

**Scheme 11:**
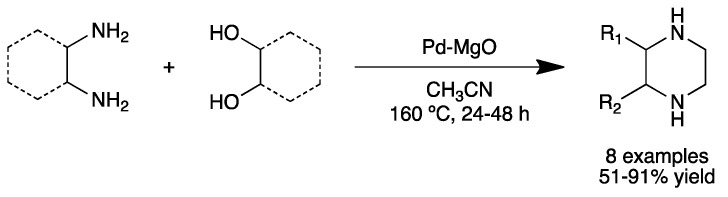
Synthesis of piperazines catalyzed by Pd–MgO [54].

Corma and co-workers were also interested in the tandem monoalkylation of amines with alcohols [54]. Employing a bifunctional PdNPs on MgO catalyst (2.5 nm crystalline PdNPs), secondary amines from both aniline and aliphatic amines and benzylic and long alkyl chain alcohols were synthesized (6 examples, 35–79% yield), where alcohols play the role of reducing agents. In situ IR studies together with ^1^H and ^13^C NMR using deuterated benzyl alcohol showed that the most probable mechanism for this reaction starts with oxidative addition of benzyl alcohol, followed by β-hydride elimination to form benzyl aldehyde. The same system was also applied to the synthesis of piperazines, building blocks used in the pharmaceutical industry (Scheme 11).

**Figure 4:**
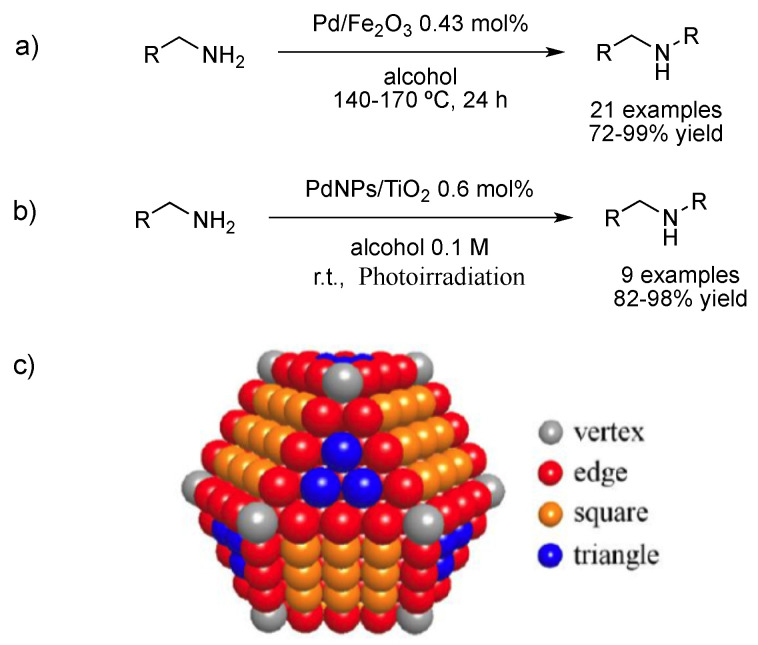
Tandem oxidation/condensation/reduction over PdNPs/Fe_2_O_3_ (**a**) [43] and PdNPs/TiO_2_ (**b**) [44]. Different types of atoms in a *fcc* nanoparticle (**c**). Reproduced from [56] with permission from the American Chemical Society.

Deng et al. prepared a large variety of secondary and tertiary amines from alcohols and amines, including anilines and benzyl alcohols (Figure 4a), catalyzed by palladium immobilized on iron oxide [55]. In fact, H_2_[PdCl_4_] and Fe(NO_3_)_3_ were reduced by co-precipitation in aqueous sodium carbonate and then calcinated to afford the catalyst, which was partially characterized, exhibiting an outstanding catalytic activity. Likewise, Hirai et al. prepared small, crystalline and highly dispersed PdNPs (ca. 2.3 nm by TEM) supported on titanium oxide (PdNPs/TiO_2_) for the *N*-monoalkylation of amines with alcohols under photochemical conditions (Figure 4b) [56]. In this case, the photoexcitation of TiO_2_ under irradiation (λ > 300 nm) promotes alcohol dehydrogenation to give aldehyde. The removed protons of alcohol are then reduced by electrons photoformed on metal particles, resulting in Pd–H. Then, imine formation catalyzed by titanium oxide and subsequent reduction by the Pd–H species present at the surface of PdNPs afforded the desired product. Interestingly, the authors determined the active sites on the nanoparticles by correlating the activity of PdNPs of different sizes with the number of each kind of atoms in an *fcc* nanoparticle (mainly four different kinds of atoms exist in a metal particle: vertex, edge, square and triangle faces; Figure 4c). They determined, by analyzing the activity of PdNPs of different sizes, that atoms on a triangle face (the number of triangle face atoms increases with PdNPs size) are the active sites for imine reduction. Nonetheless, using bigger PdNPs with a larger number of Pd on triangle faces is detrimental for the catalytic selectivity since competition between imine and alcohol for absorption on the nanoparticle became feasible.

More recently, Li et al. prepared PdNPs deposited on ZnInS_4_ via photoreduction of the palladium precursor under visible light [57]. Characterization (TEM and UV-vis) of the material showed that crystalline flower-like microspheres of ZnInS_4_ are covered exclusively with Pd(0) nanoparticles of ca. 6 nm. UV-vis spectra display absorption bands of Pd–ZnInS_4_ at 400 nm in the visible light region. Monoalkylation of amines with alcohols took place under smooth conditions (rt., Pd 0.2 mol%) to generate secondary amines in moderate to good yields, limited to the use of benzyl alcohol bearing electron-withdrawing groups. In addition, the catalyst was recycled up to three times without significant loss of activity.

**Scheme 12:**
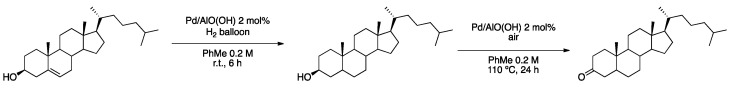
Sequential olefin hydrogenation and aerobic alcohol oxidation reaction of cholesterol to give cholestane-3-one [51].

Park et al. applied the aluminum hydroxide-supported PdNPs described above, Pd/AlO(OH), in the olefin hydrogenation of alkenes followed by aerobic alcohol oxidation for a variety of examples (88–99% yield), including the sequential reaction from cholesterol to give cholestane-3-one in 99% yield (Scheme 12) [51].

**Scheme 13:**
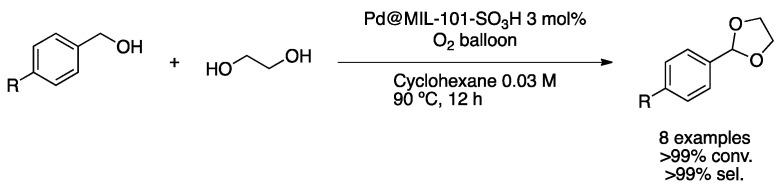
Tandem oxidation/acetylation of benzyl alcohol derivatives over Pd@MIL-101-SO_3_H [58].

Recently, Yang and co-workers prepared a multifunctional catalyst of PdNPs with both Lewis and Brønsted acid sites encapsulated within a sulfonated MOF (Pd@MIL-101-SO_3_H) [58]. The catalyst was prepared by impregnation of Pd(NO_3_)_2_ in the support and reduction under hydrogen pressure. Complete characterization of the material showed small spherical nanoparticles of crystalline Pd(0) homogeneously dispersed in the MOF, as proved by the HAADF-STEM image. XPS spectroscopy exhibited the presence of mainly Pd(0) but also the presence of Pd(II) species at the surface. The as-prepared catalyst was applied to the oxidation/acetylation reaction of benzyl alcohol derivatives with excellent conversion and selectivity (Scheme 13).

#### 2.1.3. Multi-Step Hydrodeoxygenation Reactions

**Figure 5:**
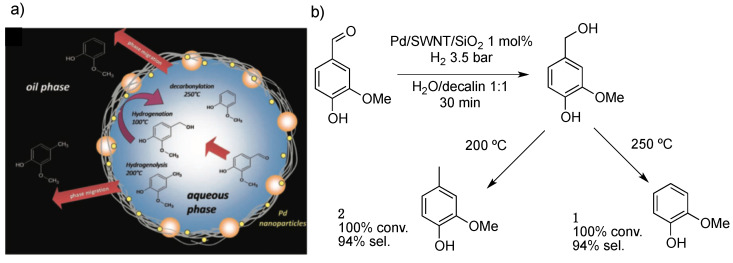
Schematic representation of the reaction at the water/oil interface in the solid stabilized emulsion (**a**). Hydrodeoxygenation reaction over Pd/SWNT/SiO_2_ (**b**) [63]. Reproduced from [63] with permission from the American Association for the Advancement of Science.

Biomass feedstocks are mixtures of biopolymers, including lignin and cellulose [59]. In contrast to fossil hydrocarbons, biopolymers are composed of oxygenated macromolecules. Especially, the structure of lignin is quite complex, making its valorization into value-added compounds by the reactivity of ether and alcohol groups very challenging [60]. Catalytic hydrodeoxygenation is the most important and feasible strategy for biofuel upgrade [61]. Vanillin (4-hydroxy-3-methoxybenzaldehyde) is a common component of pyrolysis oil derived from the lignin fraction [62]. Resasco and co-workers synthesized a catalyst capable of stabilizing water/oil emulsions at the same time that catalyzed the hydrodeoxygenation of vanillin [63]. Pd(NO_3_)_2_ was impregnated on single-walled carbon nanotubes (SWNT) supported on silica nanoparticles and then reduced to form PdNPs. Full characterization of the material showed that more defects on CNT enhanced palladium anchoring to the support surface. In fact, the material (SWNT/SiO_2_) stabilized the water/decalin emulsion, avoiding the use of surfactants that are usually difficult to separate from the final product mixture. In addition, products formed during the reaction migrated from the aqueous to the oil phase, enabling the recycling of the catalytic system (Figure 5a). Catalysis was conducted on a semi-continuous flow reactor under low hydrogen pressure (3.5 bar). Tuning the reaction temperature, the system selectivity shifted towards decarboxylation product 2-methoxyphenol 1 or hydrogenolysis product 2-methoxy-4-methylphenol 2 (Figure 5b).

Wang et al. developed a catalyst consisting of PdNPs supported on mesoporous *N*-doped carbon (Pd/CN), obtained from an ionic liquid, for the hydrodeoxygenation of vanillin [64]. TEM characterization showed small and spherical particles but showed broad size distribution. XPS analysis displayed the presence of a mixture of Pd/Pd(II). Hydrodeoxygenation reaction worked well at 150 °C but under harsher conditions than those reported by Resasco (10 bar H_2_). Xiao et al. obtained similar results under the same conditions using mesoporous sulfonated resin-supported PdNPs as catalysts [65].

**Scheme 14:**
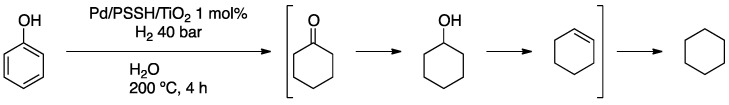
Hydrodeoxygenation of phenol over Pd/PSSH/TiO_2_.

Anderson and co-workers were also interested in the hydrodeoxygenation reaction, using PdNPs encapsulated in a polystyrene sulphonic acid and then supported on titanium oxide (Pd/PSSH/TiO_2_) [66]. Full characterization (XRD, TEM, EDX) showed a uniform distribution of 13 nm PdNPs on the support. Under quite harsh conditions (200 °C, 40 bar H_2_), hydrodeoxygenation of phenol gave high conversion, yielding cyclohexane (Scheme 14).

El-Shall et al. deeply studied the hydrodeoxygenation of vanillin over different catalysts. First, they prepared PdNPs incorporated within sulfonic acid-functionalized Cr-based MOF, obtaining a good selectivity towards 2-methoxy-4-methylphenol (Table 3. entry 1) [67]. Then, they modified the nature of the support to a Zr-based, amine-functionalized MOF, which showed a considerable improvement in the turnover frequency of the reaction under similar conditions (Table 3. entry 2) [68]. Moreover, PdNPs on a Ce-based MOF were wrapped on partially reduced graphene oxide [69]; this catalyst showed remarkable activity and selectivity towards the desired product (Table 3. entry 3).

#### 2.1.4. Multi-Step, Cross-Coupling Reactions

**Figure 6:**
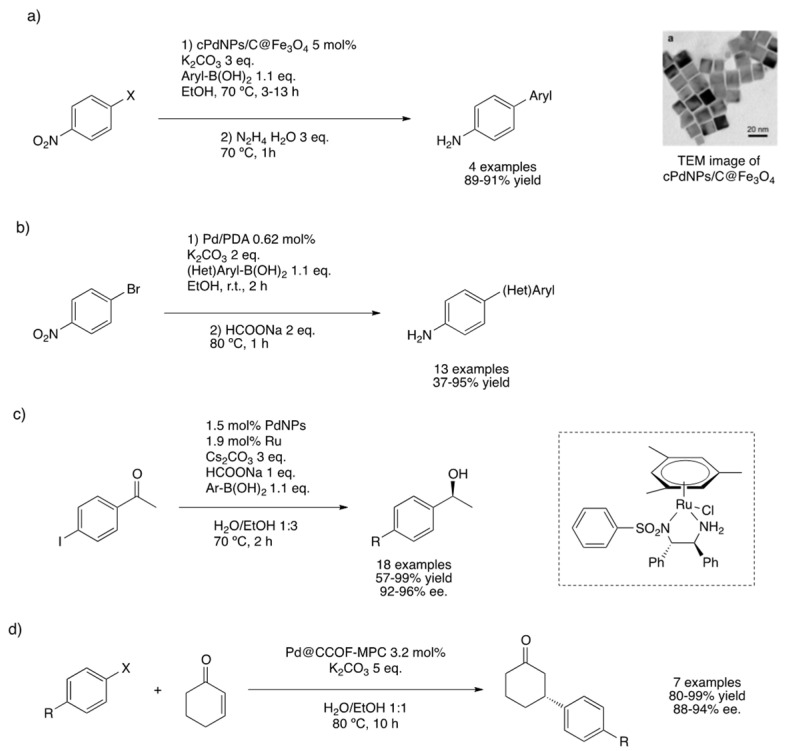
Sequential Suzuki–Miyaura cross-coupling coupled to reduction reactions over cubic PdNPs/C@Fe_3_O_4_ (**a**) [73,74], Pd/PDA (**b**) [75] and dual Pd–Ru supported on SiO_2_NPs (**c**) [77]. Tandem asymmetric reductive Heck coupling over Pd@CCOF-MPC (**d**) [78]. Reproduced from [74] with permission from the American Chemical Society.

Metal-catalyzed cross-coupling reactions are among the most powerful transformations making carbon–carbon bonds. Over the last 40 years, these methods have revolutionized the synthetic methodologies for the preparation of natural products, organic polymers or building blocks for supramolecular chemistry, among others [70]. In particular, Heck, Suzuki and Negishi were awarded the chemistry Nobel Prize in 2010 for their contributions to cross-coupling reactions in organic chemistry [71]. This kind of transformation leads to a plethora of key intermediates in the synthesis of biologically active compounds from simple entities [72]. In the context of one-pot multi-step processes, cross-couplings can be combined to hydrogenation or other couplings to obtain added-value compounds. Pitchumani’s group prepared a palladium catalyst exhibiting magnetic properties for the sequential Suzuki–Miyaura coupling (S–M)-reduction reaction [73]. Cubic crystalline PdNPs, containing an excess of {100} facets, were synthesized from Na_2_[PdCl_4_] reduced by ascorbic acid, stabilized by PVP and deposited on magnetite (C@Fe_3_O_4_) [74]. Iron chloride and urea gave nanospheres at high temperatures, constituted of an iron core (ca. 100–150 nm) and a carbon shell (ca. 20–25 nm). Full characterization (TEM, scanning electron microscopy (SEM), PXRD and XPS) of the as-prepared material showed that the smallest Pd-nanocubes (ca. 13 nm) are embedded on the surface of the support. The catalytic system performed the sequential reaction without isolation of the intermediates, leading to the Suzuki–Miyaura cross-coupling product followed by the reduction with hydrazine of the nitro group contained in the resulting biaryl (Figure 6a). Magnetic properties of the catalytic material enabled its easy separation from the liquid phase by means of an external magnet and its recycling (up to five consecutive runs without activity loss). Comparing the activity of cubic and spherical PdNPs, authors showed that turnover frequency was around two times higher using cubic than spherical nanoparticles (TOF 135 h^−1^ vs. 61 h^−1^ for S–M and 270 h^−1^ vs. 160 h^−1^ for reduction).

A similar sequential reaction was studied by London et al. using PdNPs supported on polydopamine [75]. Nanoparticles were prepared by a simple methodology, stirring metal precursor and support in MeOH at room temperature, giving small spherical nanoparticles (1–3 nm) mainly constituted of palladium oxides [76]. S–M/transfer hydrogenation sequential reaction was carried out under relatively mild conditions (in ethanol at room temperature); a large variety of examples was obtained (13 examples), including aryl and heteroaryl halides. Reduction of a nitro group into anilines was possible by transfer hydrogenation using sodium formate as a hydrogen source (Figure 6b). Liu and co-workers employed a dual catalytic system for accomplishing the S–M/transfer hydrogenation tandem reaction [77]. Actually, both PdNPs and a Ru(II) chiral complex were supported on SiO_2_ nanoparticles; using this catalyst, S–M between arylboronic acids and aryl halides bearing a ketone, followed by transfer hydrogenation using sodium formate as a hydrogen source, led to the corresponding chiral alcohols with high enantiomeric excesses (Figure 6c).

Heck cross-coupling is also an important tool in particular for the synthesis of styrene derivatives. Selective reduction of substituents in the resulting aromatic moiety without modifying the carbon–carbon double bond is a challenge and important reaction to obtain vinylaniline derivatives, which can be further functionalized. London et al. employed the same catalyst described above for the sequential S–M/transfer hydrogenation reaction [76]. The independent Heck and transfer hydrogenation reactions gave moderate to good yields using low metal loads (0.57 mol% Pd). Despite the fact that sequential reaction afforded only 38% of the desired product, reduction selectivity was very high since carbon–carbon double bond was not modified during the reaction.

**Scheme 15:**
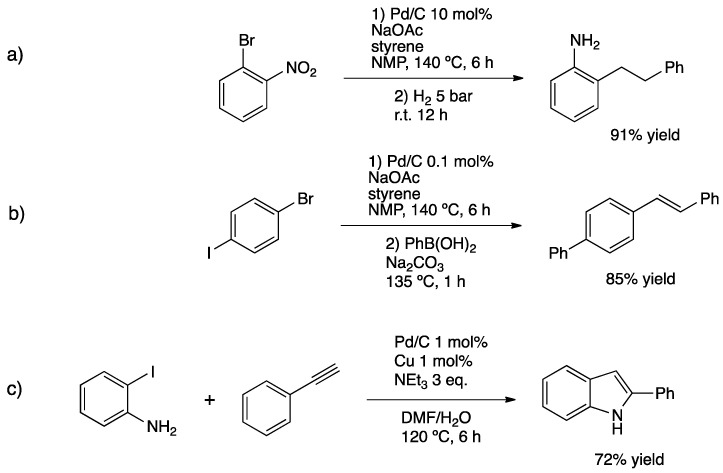
One-pot multi-step synthesis over Pd/C catalyst: Heck/hydrogenation reaction (**a**), Heck/Suzuki coupling (**b**) and tandem Sonogashira C–C coupling/cyclization process for the synthesis of 2-phenylindole (**c**) [79].

Dong and co-workers studied the asymmetric reductive Heck coupling of 2-cyclohexen-1-one with different aryl halides [78]. PdNPs were coated on a homochiral covalent organic framework (Pd@CCOF-MPC); its characterization showed that only Pd(0) was present at the surface of the support as spherical nanoparticles of around 2–5 nm. The as-prepared material gave high yields and enantioselectivity affording the corresponding (*R*)-cyclohexanones (Figure 6d). NMR experiments using D_2_O demonstrated that water was actually the hydrogen source for the transfer hydrogenation. Djakovitch et al. used commercially available Pd/C for three different one-pot multi-step processes [79]. Under harsh conditions (10 mol% Pd), they showed the feasibility of the sequential Heck/hydrogenation reaction for the synthesis of 2-(2-phenylethyl)aniline. In this case, the reduction was not selective, and both nitro group and vinyl C=C bond were reduced. The same catalytic system was employed for a sequential Heck/Suzuki cross-coupling to obtain 4-styrylbiphenyl in a high isolated yield. The selectivity is controlled because under milder conditions (0.1 mol% catalyst), only oxidative addition of the C–I bond occurred in the presence of styrene, whereas the bromide remained intact. Then, in the presence of boronic acid, S–M coupling on the C–Br bond took place. Additionally, the synthesis of 2-phenylindole via Sonogashira cross-coupling followed by intramolecular hetero-annulation was carried out (Scheme 15).

**Scheme 16:**
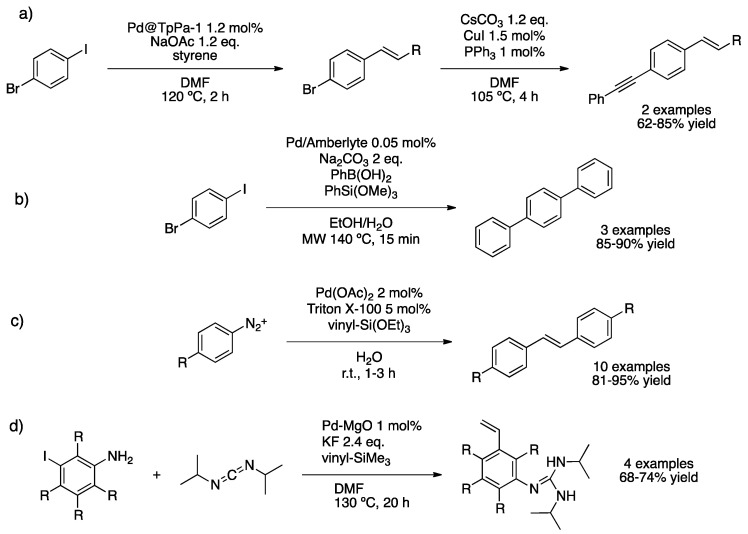
Sequential Heck–Sonogashira coupling over PdNPs@TpPa-1 (**a**) [80]. Tandem Suzuki/Hiyama coupling using Pd/amberlite catalyst (**b**) [81]. Tandem Heck/Hiyama coupling with in situ formed PdNPs (**c**) [84]. Synthesis of vinylguanidines over PdNPs@MgO (**d**) [85].

Banerjee and co-workers studied both Heck couplings to produce styrene derivatives (8 examples, 77–95% yield) and Sonogashira couplings to generate alkenylarenes (10 examples, 60–94% yield), using PdNPs immobilized on a covalent organic framework (COF TpPa-1) [80]. The catalyst was constituted of an N and O enriched sheet-like structure covalent organic framework (COF) containing well-dispersed spherical PdNPs (ca. 7 nm). This catalytic system was applied in one-pot sequential Heck/Sonogashira coupling for the synthesis of (alkynyl)(alkenyl)arenes. In fact, Heck reaction of aromatic dihalide (1-bromo-4-iodobenzene) with both aliphatic and aromatic olefins in the presence of PdNPs@TpPa-1 was followed by Sonogashira reaction using phenylacetylene (Scheme 16a). Kaur and co-workers proposed a similar process for the tandem Suzuki/Hiyama coupling [81]. Hiyama cross-coupling enables the synthesis of biaryls by using silane derivatives [82]. For this purpose, PdNPs encapsulated on Amberlite resin (crosslinked polystyrene) were prepared, obtaining small spherical (3–10 nm) nanoparticles exhibiting an *fcc* Pd(0) structure [83]. This catalyst permitted the formation of terphenyls under micro-wave irradiation reaching high yields in only 15 min (Scheme 16b). Catalyst recycling was successful for five runs without metal leaching. Kamble’s group investigated the tandem Heck/Hiyama coupling using PdNPs formed in situ; Pd(OAc)_2_ and polymeric surfactant led to the formation of nanoparticles in water at room temperature [84]. Heck reaction from diazonium salts and vinylsilanes led to the synthesis of styrene derivatives bearing a silyl group that could be further functionalized to give stilbenes (Scheme 16c). Corma’s group applied the PdNPs@MgO catalyst in the synthesis of vinylguanidines from iodoanilines through tandem aniline–carbodiimide coupling and Hiyama cross-coupling on the iodo-position, at 130 °C (Scheme 16d) [85].

**Scheme 17:**
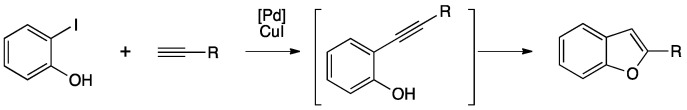
General synthesis of benzofurans via tandem Sonogashira coupling/5-*endo*-*dig* cyclization.

The synthesis of heterocycles is of great interest due to their occurrence in nature and their biological activities. In particular, benzofurans are useful as anti-tumor agents and in the treatment of type 2 diabetes [86]. Generally, the preparation needs several steps, but the synthesis can be accomplished through a tandem Sonogashira coupling/5-*endo*-*dig* cyclization (Scheme 17).

**Scheme 18:**
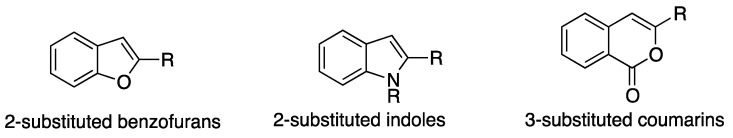
Heterocycles synthesized using the commercially available heterogeneous Pd/C catalyst [87,88,89].

Pal et al. used commercially available Pd/C to obtain 2-substituted benzo[b]furans from iodophenols [87]. Aliphatic and aromatic 2-substituted benzofurans were prepared using CuI as co-catalyst under relatively mild conditions (10 mol% Pd in water at 80 °C for 3 h). The same methodology starting from iodoanilines afforded aryl and alkyl-substituted indoles, while starting from iodobenzoic acid enables the synthesis of 3-substituted isocoumarins (Scheme 18) [88,89].

**Scheme 19:**
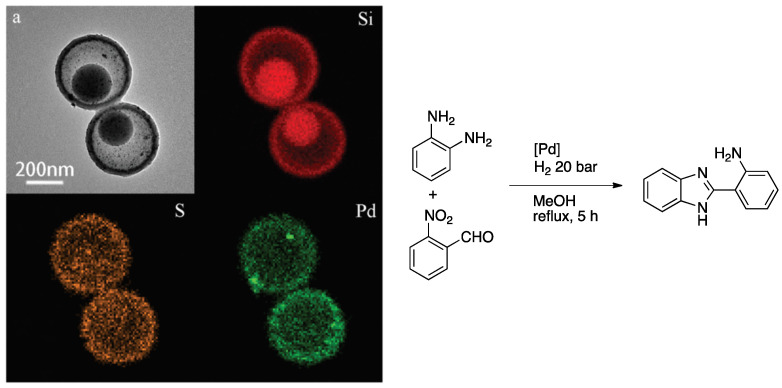
Left: TEM image (a) and elemental mapping (TEM coupled to EDS analysis) of pomegranate-like Pd–silica yolk–shell structure. Right: synthesis of 2-(2-aminophenyl)-1*H*-benzimidazole using pomegranate-like Pd–silica yolk-shell structure as catalyst [90]. Figure on the left reproduced from [90] with permission from the Royal Society of Chemistry.

Huo et al. synthesized a mesoporous silica yolk–shell pomegranate-like structure where PdNPs were dispersed within the shell of the nanomaterial [90]. A silica core was covered by a thiol nanorattle, and PdNPs were embedded inside the material. Mesoporous silica improved the diffusion of the substrates, while −SO_3_H groups gave the acidic conditions necessary for the reaction to proceed. EDS elemental mapping showed that both palladium and sulfur are present at the surface of the material. XPS spectroscopy showed that 60% of Pd in the catalyst was present as zero-valent metal, while the rest corresponded to Pd(II). Synthesis of benzimidazole derivatives was catalyzed by this material through three steps: amine–aldehyde condensation, followed by cyclization and then reduction of nitro group, reaching acceptable TOF (6.2 h^−1^) (Scheme 19).

**Figure 7:**
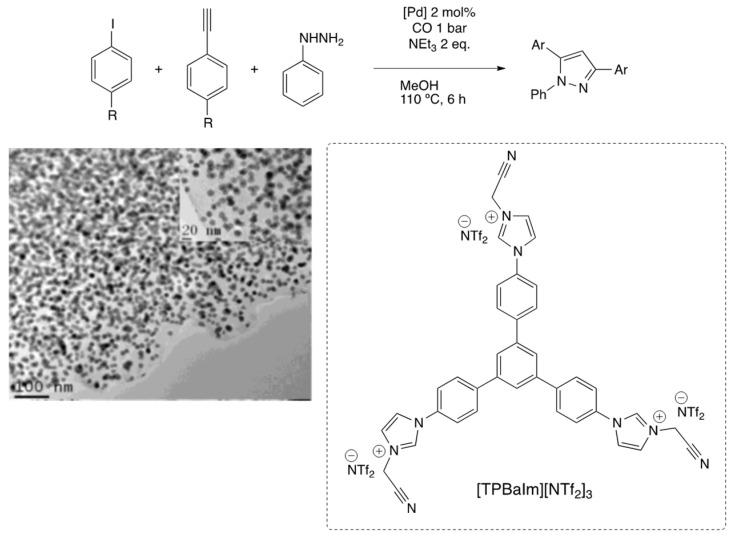
TEM image of Pd@CN and four-component synthesis of triarylpyrazoles [91]. Reproduced from [91] with permission from the American Chemical Society.

Li and co-workers developed a tandem reaction for the synthesis of triaryl-substituted pyrazoles using PdNPs supported on *N*-dopped carbon (Pd@CN) [91]. Pd(OAc)_2_ and tridentate NHC-ligand ([TBPAIm][NTf_2_]_3_) were carbonized under nitrogen atmosphere to afford well-dispersed small spherical PdNPs (12.3 ± 1.1 nm) (Figure 7). PXRD showed the presence of crystalline nanoparticles of mainly Pd(0). The four-component synthesis of pyrazoles from iodoarene, phenylacetylene and phenylhydrazine under a CO atmosphere gave excellent yields (81–92% yields in 6 h).

**Scheme 20:**
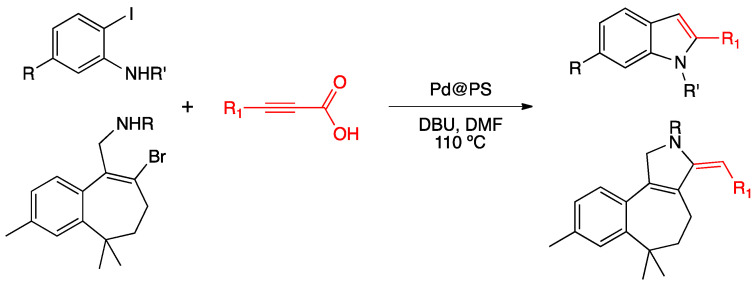
Synthesis of indoles and pyrrolines using PdNPs@PS as catalyst [92].

Das, Kumar and co-workers used PdNPs impregnated on Amberlite (PS) for the synthesis of indoles and 3-pyrrolines through a tandem decarbonylative Sonogashira coupling/cyclization (Scheme 20) [92].

Samorjai and co-workers reported the dehydrogenation/C–H arylation tandem reaction catalyzed by PdNPs supported on Santa Barbara Amorphous-15 silica material (SBA-15) for the preparation of indoles [93]. Small PdNPs (1.5 ± 0.3 nm) stabilized by Crooks 4th generation dendrimers were supported on mesoporous silica SBA-15 [94]; this catalyst was engaged to the one-pot quantitative synthesis of 2-phenylindole from indoline. Yang et al. were interested in the synthesis of benzofurans using PdNPs supported on *N,O*-dopped hierarchical porous carbon obtained from Bamboo shoots [95]; TEM images showed spherically supported PdNPs of ca. 13 nm together with some agglomeration; this catalyst permitted to obtain more than 20 benzofuran derivatives in good yields showing higher selectivity than Pd over carbon, magnesium oxide or alumina.

**Scheme 21:**
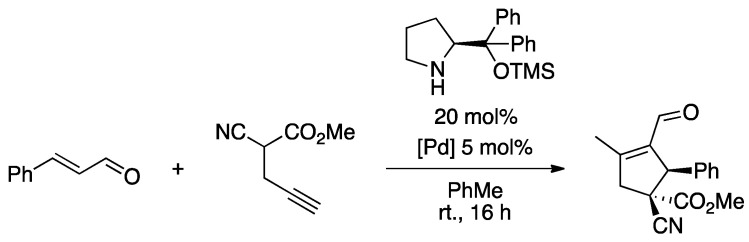
Pd-catalyzed stereoselective cascade carbocyclization [96].

Córdova and co-workers studied a stereoselective cascade carbocyclization transformation [96]. PdNPs supported on amine-functionalized rice-husk-derived silica were prepared, giving well-dispersed particles and showing high activity for Suzuki–Miyaura coupling, oxidation of alcohols and carbocyclization reaction with excellent stereoselectivity (27:1) in the presence of a chiral amine (Scheme 21).

**Scheme 22:**
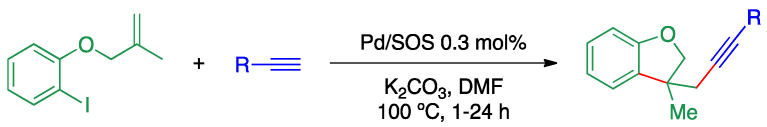
Pd-catalyzed carbocyclization over PdNPs supported on SOS [97].

Subrahmanyam et al. prepare dehydrobenzofuran derivatives from 1-iodo-2-((2-methylallyl)oxy)benzene and phenylacetylene catalyzed by PdNPs on a nanosilica material (Scheme 22) [97]. Small PdNPs (ca. 2.2 nm) were prepared from PdCl_2_ by reduction with sodium borohydride and supported on nanosilica on microsilica (SOS).

#### 2.1.5. Miscellaneous

**Scheme 23:**
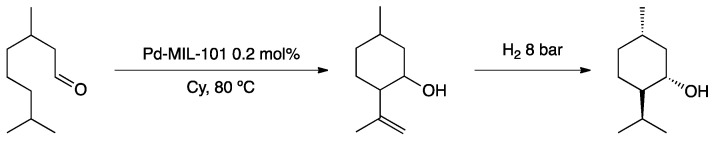
One-pot synthesis of (−)-menthol over Pd–MIL-101 [98].

In this section, we enlist some other interesting multi-step transformations involving PdNPs. Corma’s group reported a simple pathway for obtaining (−)-menthol, a compound widely used in the food and pharmaceutical industries that is generally obtained through multi-step reactions from thymol or myrcene [98]. They developed a one-pot synthesis from citronellal via cyclization and hydrogenation steps (Scheme 23). PdNPs were impregnated on a MOF containing coordinatively unsaturated Cr(III) cations. TEM micrographs of the material showed the presence of small spherical PdNPs (ca. 3 nm) and no modification of the support after impregnation. Under a sequential methodology, the desired product was obtained with good chemo- and diastereoselectivity (81%).

Recently, Wu et al. prepared a bifunctional catalyst consisting of mesoporous silica nanoparticles (MSN) loaded with both PdNPs and *Candida antartica* lipase B (CalB) [99]. This material was also tuned by surface alkylation resulting in a tailor-made particle hydrophobicity, which permitted to disperse the catalyst in organic solvents. Full characterization was conducted, proving the homogeneous dispersion of both PdNPs and CalB in the surface of MSN. Interestingly, TEM images together with wide-angle XRD showed very small crystalline *fcc* PdNPs with a narrow size distribution (1.8 ± 0.6 nm). The as-prepared catalyst was applied to the sequential hydrogenation/transesterification reaction of benzaldehyde with ethyl hexanoate to produce benzyl hexanoate in good yield (76%). Moreover, the catalytic system was recycled at least five times without observing an agglomeration of nanoparticles or palladium leaching.

Marks and co-workers were interested in the ether C–O bond hydrogenolysis via a dual catalytic system where lanthanide triflate-catalyzed C–O bond scission, followed by Pd-catalyzed hydrogenation [100]. PdNPs were prepared by reduction of palladium hexafluoroacetylacetonate (Pd(hfac)_2_) with formaldehyde, giving small nanoparticles (1.1–2.9 nm) that were deposited on alumina via atomic layer deposition [101]. The catalytic reaction in ionic liquid (1-ethyl-3-methylimidazolium triflate, [EMI][OTf]) under 41 bar H_2_, enabled the preparation of primary alcohols and phenols from linear and cyclic ethers with high selectivity. Recently, Lin et al. prepared a mixed-ligand MOF with both strong Lewis acid sites and PdNPs, obtained from the reduction of [PdCl_2_(CH_3_CN)_2_] under H_2_ (20 bar) [102]. The as-prepared material was highly active for tandem dehydroalkoxylation–hydrogenation of ethers, alcohols and esters to form saturated alkanes under smooth conditions by C–O bond cleavage (10 examples, 61–99% yields). The catalyst was reused six times.

Li et al. prepared PdNPs on ZnO supported on biomass-derived zeolites [103]. The resulting catalytic material was employed for CO_2_ hydrogenation to give dimethyl ether, where Pd/ZnO catalyzed the conversion of carbon dioxide into methanol and the bio-zeolite catalyzed methanol dehydration to dimethyl ether, obtaining a promising 10% conversion and 31% selectivity.

**Scheme 24:**
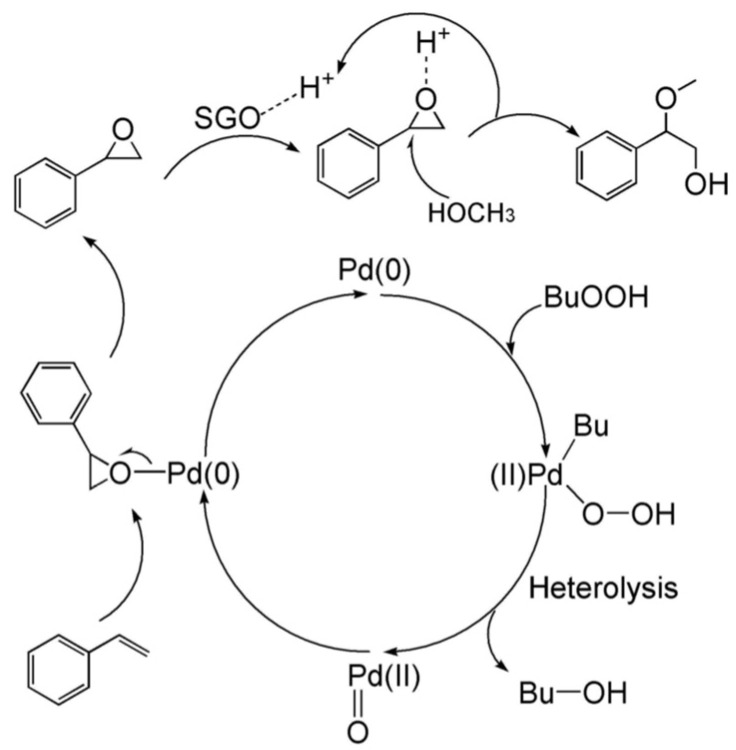
Proposed mechanism of epoxidation/hydromethoxylation tandem process catalyzed by PdNPs@MIL-101@SGO [104].

Han and co-workers prepared PdNPs encapsulated in chrome-based metal–organic framework (MIL-101) on sulfonated graphene oxide (SGO) [104]. The novel material was fully characterized, showing the formation of well-dispersed spherical nanoparticles (ca. 3.5 nm) and applied to tandem epoxidation/hydroxymethoxylation of styrene using t-butylhydroperoxide (TBHP) as oxidant. TBHP oxidized PdNPs forming Pd=O species, which epoxidized styrene to styrene oxide. The epoxide was further protonated by sulfonated graphene oxide and reacted with methanol by a selective nucleophilic addition to give the corresponding trans-β-alkoxy alcohol (Scheme 24).

**Scheme 25:**
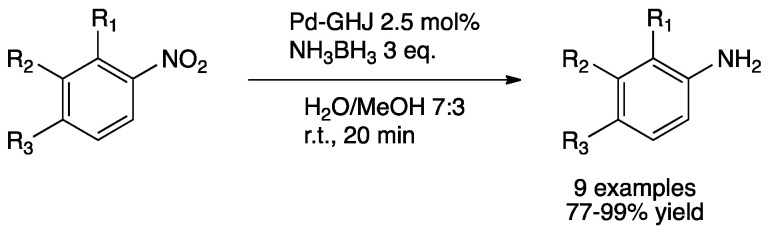
Tandem dehydrogenation of ammonia–borane and transfer hydrogenation of nitroarene catalyzed by Pd–GHJ [105].

Metin et al. investigated the tandem dehydrogenation of ammonia–borane adduct followed by transfer hydrogenation of nitroarenes catalyzed by PdNPs supported on graphene hydrogel (Pd–GHJ) [105]. Ammonia–borane is an effective hydrogen source exhibiting a high solubility in common solvents [106]. Thus, PdNPs catalyzed first the release of hydrogen from ammonia–borane and then the reduction of the nitroarene. PdNPs, synthesized by polyol method, showed the exclusive formation of Pd(0) species as confirmed by XPS. With this catalyst, aniline derivatives were prepared under mild conditions (water/methanol solvent mixture at room temperature for 20 min) (Scheme 25).

Recently, Chen et al. prepared PdNPs supported on aluminum-based metal–organic framework MIL-53(Al) into CeO_2_ nanotubes [107]. This catalyst gave high activity in the tandem dehydrogenation of ammonia–borane and semi-hydrogenation of phenylacetylene, reaching up to 96% selectivity of styrene in just 1 min at room temperature and atmospheric pressure.

### 2.2. PdNPs Dispersed in a Liquid Phase

#### 2.2.1. Multi-Step Condensation/Reduction Reactions

As stated above, direct reductive amination from alcohols, aldehydes or ketones is of great importance for obtaining functionalized amines. Devi et al. used PdNPs stabilized by a natural polysaccharide, gum acacia, to prepare secondary amines under mild conditions (in methanol at room temperature under 1 bar of H_2_) [108]. Gum acacia plays a double role in the synthesis of MNPs, as a stabilizer as well as a reducing agent of metal precursors. TEM analyses showed well-dispersed 9 nm spherical nanoparticles, exhibiting a crystalline *fcc* structure of Pd(0) (determined by PXRD), while XPS showed the presence of both Pd(II) and Pd(0). This catalyst was efficient for the synthesis of aryl and alkyl-substituted amines but limited to arenes bearing electron-donating groups. Pucheault and co-workers synthesized PdNPs embedded in tetrabutylammonium bromide, an ionic liquid that is solid at room temperature [109]. TEM, together with a selected area diffraction pattern, showed the formation of small particles with narrow size distribution (4.0 ± 0.3 nm) and high crystallinity. This colloidal-supported catalyst, liquid at high temperature (>140 °C), was applied in the direct reductive amination from benzyl alcohol derivatives, with amines obtaining the expected secondary amines in the absence of molecular hydrogen.

**Figure 8:**
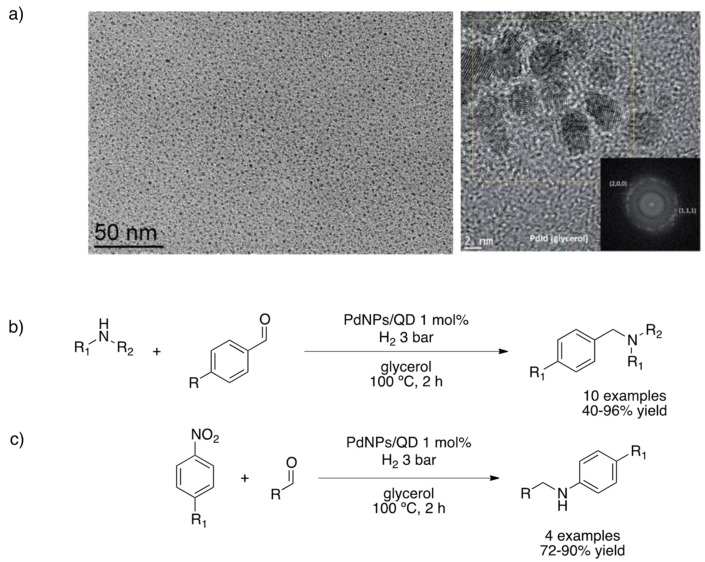
TEM (top left) and HR-TEM (top right) images of PdNPs stabilized with quinidine in glycerol (**a**). Synthesis of secondary amines (**b**) and *N*-substituted anilines (**c**) under hydrogen pressure catalyzed by PdNPs/QD in glycerol [114]. Reproduced from [114] with permission from the Royal Society of Chemistry.

Gómez and co-workers have vast experience in the synthesis and catalytic applications of metal nanoparticles in glycerol, where the solvent plays a key role as liquid support, enabling an efficient immobilization of metal-based NPs and easy recycling of the catalytic phase [110,111,112,113,114,115]. Therefore, PdNPs stabilized by cinchona-based alkaloids were prepared for the tandem synthesis of functionalized amines via reductive amination of aldehydes [114]. These PdNPs were synthesized in neat glycerol by metal precursor decomposition under a hydrogen atmosphere using cinchona-derivatives as stabilizers. The resulting black colloidal solution was fully characterized. (HR)-TEM images taken directly from the glycerol solution (enabled given its negligible vapor pressure) showed very small, spherical and well-dispersed nanoparticles (1.4 ± 0.3 nm) with a crystalline *fcc* structure, as confirmed by PXRD (Figure 8a). Elemental analysis, together with the determination of hydride contents by alkene titration (quantified by ^1^H NMR), permitted to determine an idealized molecular formula Pd_147_(QD)_61_H_74_. The as-prepared material was very efficient for the synthesis of benzylamines, from benzaldehydes and amines, including aqueous ammonia (Figure 8b). The same methodology was applied starting from nitrobenzene to provide *N*-substituted anilines (Figure 8c). This catalytic system was easily recycled four times; further catalyst regeneration with H_2_ allowed recovering catalyst activity.

#### 2.2.2. Multi-Step Cross-Coupling/Reduction Reactions

Our group was also interested in palladium-catalyzed cross-coupling reactions and their applications in multi-step processes. PdNPs obtained from the reduction of [Pd_2_(dba)_3_] in imidazolium-based ionic liquids (IL) under hydrogen pressure were used in the sequential Heck coupling/hydrogenation reaction, in particular for the synthesis of 4-(4-methoxyphenyl)butan-2-one, industrially used as raspberry scent (Table 4) [116]. Moreover, PdNPs in IL were immobilized on different supports, such as silica and functionalized multi-walled carbon nanotubes [117]. The as-prepared supported-ionic liquid phase (SILP) catalytic material gave high conversion for the sequential Heck/hydrogenation reaction permitting to recycle the catalyst for at least four runs. Furthermore, PdNPs in [EMI][MeHPO_3_] stabilized by phosphines resulted in small and very well-dispersed nanoparticles, as observed by TEM (analyses of the IL phases) [118]. In this case, PdNPs acts as a reservoir of catalytically active palladium molecular species. The reduction of the exocyclic C=C double-bond occurred without the need of external molecular hydrogen, strongly suggesting a hydrogen-transfer reaction catalyzed by a Pd–hydride species formed during molecular-type Heck coupling, thanks to the basic behavior of the IL anion, methyl hydrogenphosphonate.

**Figure 9:**
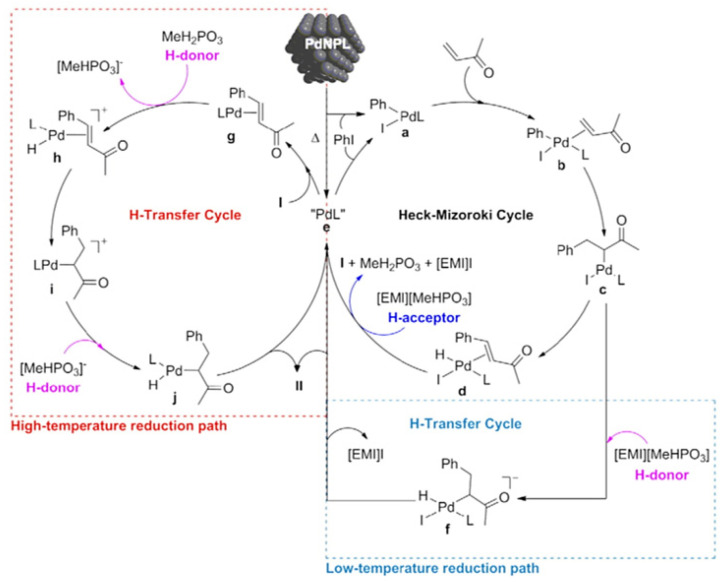
Proposed mechanism for the one-pot sequential Heck/hydrogenation reaction promoted by pre-formed PdNPs in [EMI][MeHPO_3_] (**I** stands for 4-phenyl-3-buten-2-one and **II** stands for 4-phenyl-2-butanone). Reproduced from [119] with permission from Elsevier.

Later, we were interested in how to design an ionic liquid with the aim of promoting the transfer hydrogenation, assisted by PdNPs [119]. PdNPs stabilized with thioether-phosphine ligands were prepared in [EMI][MeHPO_3_]. TEM analysis showed the formation of spherical nanoparticles (4.0 ± 0.3 nm) dispersed in the ionic liquid when the thioether-phosphine ligand contained aromatic groups, while ligands bearing alkyl chains led to some agglomerates together with well-dispersed nanoparticles. Interestingly, when the reaction was carried out under hydrogen pressure at low temperature, the reduction to 4-phenyl-butan-2-one was inhibited by the presence of thioether-phosphine ligands. By comparison to other ionic liquids, it was proven that [EMI][MeHPO_3_] was actually playing the role of hydrogen source at high temperature; at low temperatures, it behaves as a hydrogen acceptor (Figure 9).

As shown, ionic liquids have proven their ability to stabilize colloidal systems, in particular those based on imidazolium salts. However, their controversial environmental impact together with their high cost compelled us to search for environmentally friendly solvents. In this context, glycerol appears as an elegant alternative coming from biomass and produced in tons as a waste in the biodiesel industry. Due to its low cost, non-toxicity, negligible vapor pressure and high solubilizing ability of both organic and inorganic compounds, glycerol constitutes a good candidate to immobilize catalysts. In fact, its hydroxy groups can trigger supramolecular arrangements by hydrogen bonding, enabling good nanoparticles dispersion [120,121]. We studied the immobilization of PdNPs in glycerol and proved that additional stabilizers were necessary to avoid the formation of agglomerates. Glycerol-soluble phosphines as triphenylphosphine-3,3′-3′′-trisulfonic acid trisodium salt (TPPTS) and PTA-based ammonium salts (PTA = 1,3,5-triaza-7-phosphaadamantane) were suitable for stabilizing metal nanoparticles in glycerol [122,123].

**Scheme 26:**
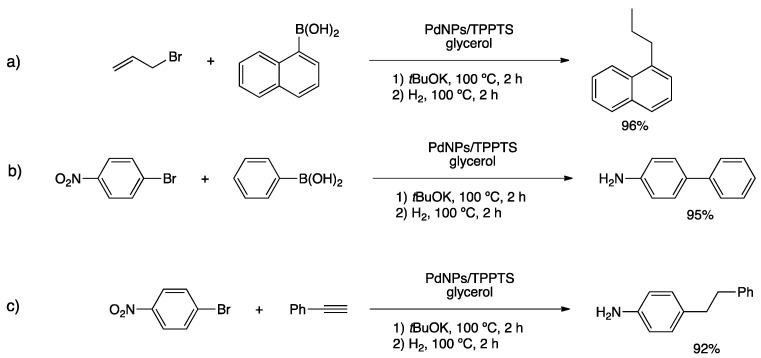
One-pot cross-coupling/hydrogenation reactions catalyzed by PdNPs/TPPTS in glycerol (**a**,**b**,**c**) [122].

**Figure 10:**
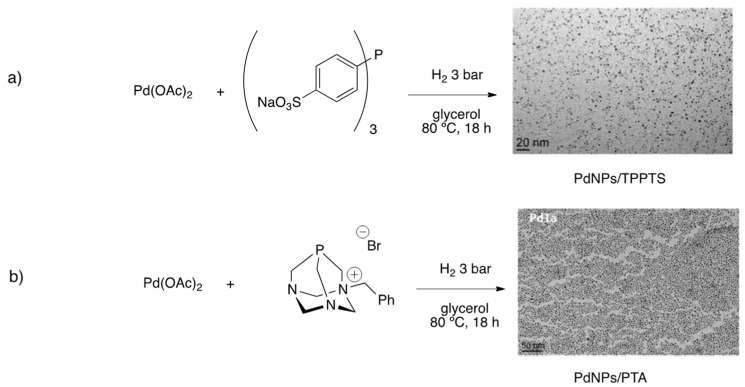
Synthesis of PdNPs in glycerol stabilized by different ligands: TPPTS (**a**) and PTA (**b**) [122,123]. Reproduced from [122] with permission from Wiley and from [123] with permission from Elsevier.

PdNPs from Pd(OAc)_2_ were synthesized in glycerol under hydrogen pressure and in the presence of TPPTS [122]. The as-prepared nanoparticles were fully characterized, obtaining small, spherical and well-dispersed particles (2.0 ± 0.6 nm) (Figure 10a). HR-TEM, together with PXRD analyses, confirmed the crystalline *fcc* structure of PdNPs, and EDX analysis pointed to the presence of phosphine at the metal nanoparticle surface. PdNPs in glycerol were applied in catalysis showing an outstanding performance of a variety of transformations. In fact, Suzuki–Miyaura, Heck and Sonogashira couplings gave excellent yields in short times (less than 2 h). Moreover, PdNPs/TPPTS in glycerol were able to catalyze hydrogenation reactions of several functional groups, permitting to carry out one-pot cross-coupling/hydrogenation reactions with excellent isolated yields (Scheme 26). Crucially, immobilized PdNPs in glycerol were recycled at least 10 times without losing activity nor leaching metal.

The effect of PTA derivatives as stabilizers for PdNPs in glycerol was also studied [123]. Employing a similar methodology for the synthesis of nanoparticles, a highly homogeneous dispersion of PdNPs (3.2 ± 0.8 nm) was obtained (Figure 10b). Isolated nanoparticles at solid state were analyzed by PXRD, showing the crystalline *fcc* structure of Pd(0). Comparable results for both cross-couplings and hydrogenations were obtained using PdNPs stabilized by TPPTS and PTA-ammonium salts, and recycling was also successful. However, one-pot S–M/hydrogenation reaction was less efficient under the same conditions, giving only a 54% yield of 4-phenylaniline.

**Scheme 27:**
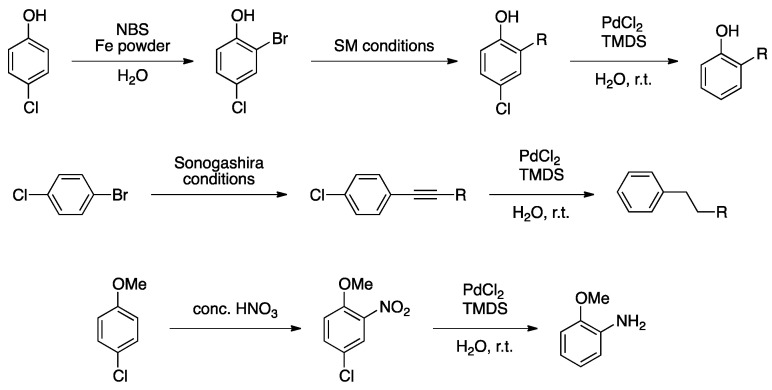
One-pot cross-coupling/reduction reactions in water catalyzed by in situ generated PdNPs [124].

Other groups were also interested in palladium-catalyzed cross-coupling reactions followed by reduction. Lipshutz and co-workers developed an efficient system for hydrodehalogenation [124]. In situ formation of PdNPs, resulting from the reduction of PdCl_2_ with tetramethyldisiloxane (TMDS) in water, was demonstrated by XPS, showing the exclusive presence of Pd(0) species. Actually, TMDS reduces Pd(II) into Pd(0), forming a molecule of H_2_, which is responsible for hydrodehalogenation under mild conditions (r.t., 10 min). This was applied to sequential processes, where the first reaction was directed by the presence of a halide that was then removed in a second step (Scheme 27). PdNPs dispersed in water were recycled four times.

**Scheme 28:**
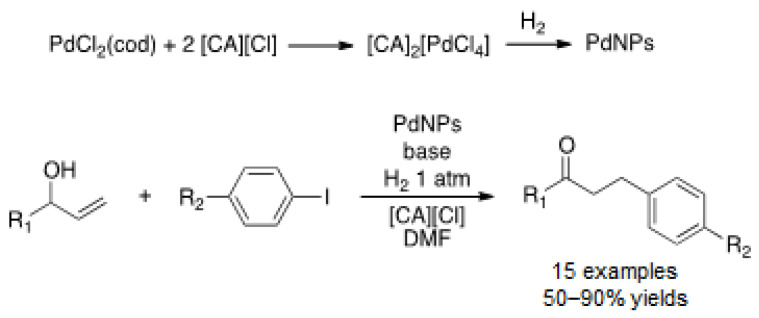
Heck reductive coupling from allylic alcohols catalyzed by pre-formed PdNPs in DMF [126].

Lovelock et al. prepared PdNPs stabilized by a phosphine immobilized in an ionic liquid polymer (Pd@PIILP) which were applied in a sequential process, S–M coupling of aryl halides bearing nitro group followed by hydrogenation, to afford aniline derivatives [125]. The as-prepared PdNPs were constituted of a mixture of Pd(0)/Pd(II) species, as shown by XPS. This catalyst was highly active for the two-step reaction affording the desired anilines in high yields (91–93%). Unfortunately, recycling was unsuccessful. Trzeciak and co-workers synthesized PdNPs from a palladium complex with imidazolium cations (CA) under hydrogen pressure [126]. TEM images displayed big particles, ca. 65 nm. This catalyst was engaged in a Heck coupling reaction of allylic alcohols with iodoarenes to give the corresponding saturated ketones under hydrogen pressure (Scheme 28).

**Scheme 29:**
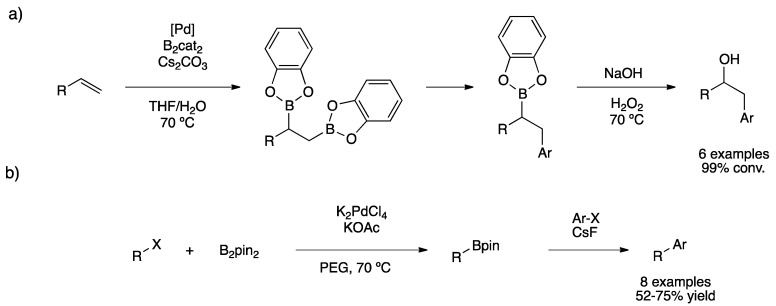
Heck reductive coupling from allylic alcohols (**a**) [127]. One-pot borylation/S–M coupling (**b**) [128].

Fernández and co-workers synthesized palladium molecular complexes for the diboration of alkenes [127]. During this study, they realized that bis(catecholato)diboron (B_2_cat_2_) favored the reduction of palladium species, forming in situ PdNPs as proven by TEM micrographs (6.9 ± 3.0 nm). Taking advantage of this behavior, they performed a one-pot diboration/Suzuki coupling, obtaining satisfactory results for the monoarylation of primary alkylboronic esters, which led to the corresponding alcohol after the oxidative workup (Scheme 29a). For the same reactivity, Sarkar’s group applied Pd nano-catalysts in one-pot sequential borylation/S–M coupling to prepare unsymmetrical biaryls and diarylmethanes from haloarenes [128]. PdNPs (ca. 6 nm) were prepared in situ by thermal decomposition of K_2_[PdCl_4_] in polyethyleneglycol (PEG). Using KOAc as a base, PdNPs catalyzed borylation of aryl and benzylhalides from both chloro- and bromo-derivatives. The addition of a base such as CsF and the haloarene to the resulting borylated compound, favored the Suzuki–Miyaura cross-coupling (Scheme 29b). This strategy permitted the preparation of different biaryls and diarylmethanes in moderate to good yields using a relatively low metal load (2–3 mol%).

Muller and co-workers were inspired by the nanoparticles synthesis early reported by Llorca, consisting of PdNPs stabilized by alkanethiolates and functionalized with organometallic fragments [129,130]. PdNPs containing a Pd(II) complex at the end of the thiol chain were employed for the one-pot hydrovinylation/S–M coupling [130]. 4-Bromostyrene and ethene formed the corresponding butenyl benzene that could be further functionalized via a Suzuki–Miyaura cross-coupling. NaBARF was needed to improve the low hydrovinylation yield, acting as a halide scavenger.

**Scheme 30:**
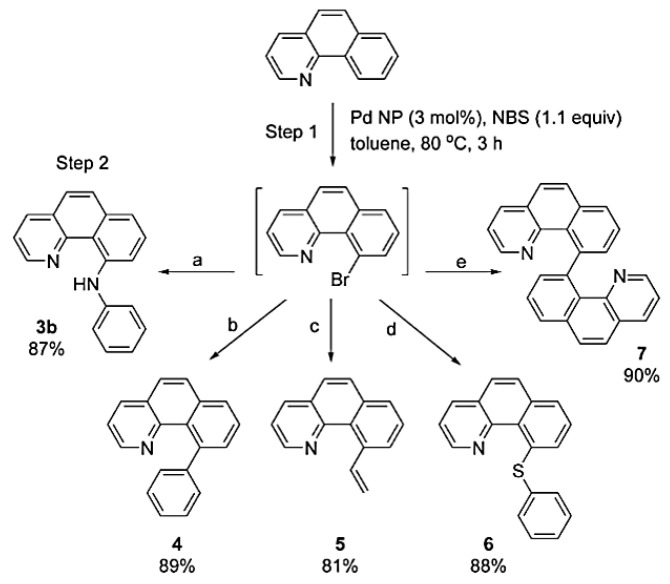
Sequential C–H bromination/cross-coupling reaction over PdNPs [131].

Kim and co-workers studied the C–H bromination/cross-coupling sequential catalysis [131]. The controlled oxidation state of palladium in the PdNPs enabled the C–H activation via Pd(IV) species and cross-coupling via Pd(0) species. PdNPs (ca. 3.5 nm) were prepared by thermal decomposition of Pd(acac)_2_ in the presence of trioctylphosphine (TOP) and oleylamine. In the presence of PhICl_2_, nanoparticles were oxidized, forming high-valent Pd(IV) species as demonstrated by XPS spectroscopy, where the Pd 3d_5/2_ peak shifted from 335.8 eV to 338.2 eV after oxidization [131,132]. Treatment with H_2_ regenerated Pd(0) nanoparticles and no modification on the mean size was observed. Extended X-ray Absorption Fine Structure (EXAFS) analysis exhibited the coordination number after oxidation and reduction. Pristine PdNPs showed a coordination number of Pd–Pd around 5, which diminished to 1 after oxidation, while coordination numbers of Pd–O and Pd–Cl increased from 1 to 3, pointing to the formation of polymeric -Pd(IV)Cl_3_O- clusters. After regeneration, the coordination number of Pd–Pd increased again to almost 7 due to the formation of Pd(0)NPs. Direct C–H activation/cross-coupling catalyzed by PdNPs gave outstanding results for a variety of processes such as Buchwald–Hartwig, Suzuki-Miyaura and Stille cross-couplings, C–S bond formation and homocoupling (Scheme 30).

**Scheme 31:**
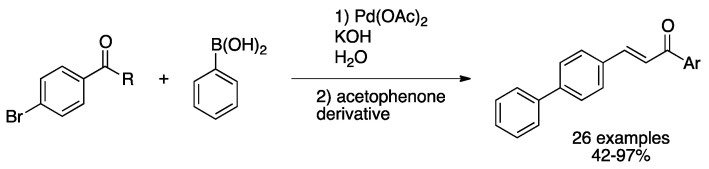
Sequential S–M/aldol-condensation reaction catalyzed by in situ formed PdNPs [133].

Tagliavini and co-workers studied the S–M coupling/aldol-condensation, an often-used synthetic methodology for final step functionalization aiming alkaloids, fungal metabolites or other natural products with biological activities [133]. PdNPs obtained in situ by reduction of Pd(OAc)_2_ in aqueous phase and stabilized by a biomass-derived surfactant catalyzed sequential reaction for the synthesis of chalcones. With this catalytic system in hand, authors carried out the sequential Suzuki–Miyaura cross-coupling followed by aldol-condensation starting from both (hetero)aryl aldehydes and ketones in water, showing great activity and robustness, as several functional groups were tolerated (Scheme 31). In addition, stabilized PdNPs were successfully recycled for at least five runs without loss of activity.

#### 2.2.3. Synthesis of Heterocycles

**Scheme 32:**
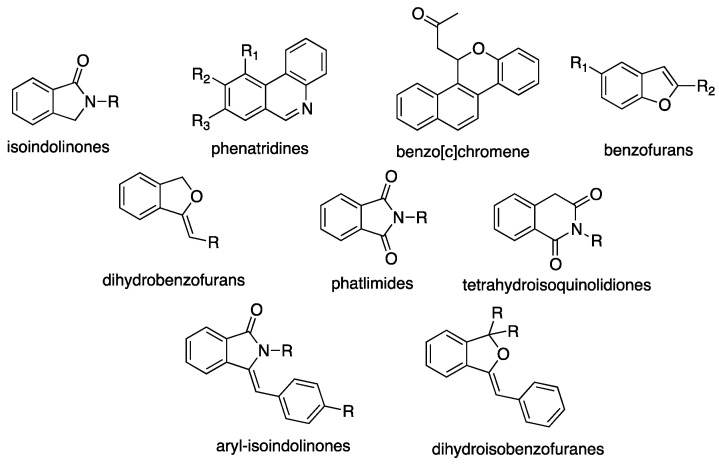
Selected heterocyclic compounds synthesized by Pd-catalyzed multi-step processes [134].

As discussed above, heterocycles are building blocks commonly found in natural and biologically active compounds as well as in commercialized drugs (Scheme 32).

Keep et al. provided a facile strategy for the synthesis of PdNPs from pre-formed palladacycle complexes under carbon monoxide atmosphere [134]. These PdNPs were applied in the tandem three-step synthesis of isoindolinones under smooth conditions (1 bar CO, r.t.) [135]. First, the nucleophilic substitution of iodobenzylbromide by the primary amine to form a secondary benzylamine derivative took place; then, carbonylation through palladium oxidative addition of the Ar–I bond and subsequent cyclization afforded substituted isoindolones in moderate to good yields (8 examples, 33–85% yields). Interestingly, conversion to isoindolinones correlated with the pKa of the primary amines used, pointing to the nucleophilic substitution is involved in the rate-determining step.

Phenanthridines are present in the structure of several natural products with biological activity, showing pharmacological effects as antifungal, antibacterial and antitumoral agents [136]. Ray and co-workers developed a methodology for the synthesis of phenanthridine derivatives via S–M coupling/Michael addition reaction catalyzed by Pd(OAc)_2_ in water in the presence of a base [137]. Despite the lack of characterization of their catalytic system without proving the actual presence of nanoparticles, authors relied on the work of Ranu et al., where palladium precursor with the same base but in the presence of sodium dodecyl sulfate in water formed PdNPs with an average size of 6–8 nm [138]. Ray and co-workers astonishingly proposed that K_3_PO_4_ can play the role of a stabilizer when tetrabutylammonium bromide (TBAB), present in the reaction mixture, is much more likely to play the role of surfactant. Nevertheless, this catalytic system turned to be effective for the synthesis of phenanthridines and benzophenanthridines, providing eight examples with moderate to good yields but using a relatively high metal load (10 mol% of Pd). Authors were also interested in the synthesis of benzo[c]chromene derivatives present in several natural products, for instance, in Cannabinol [139,140]. The same methodology via S–M/Michael addition and using in situ generation of uncontrolled PdNPs afforded eight examples in good yields (72–90%).

**Scheme 33:**
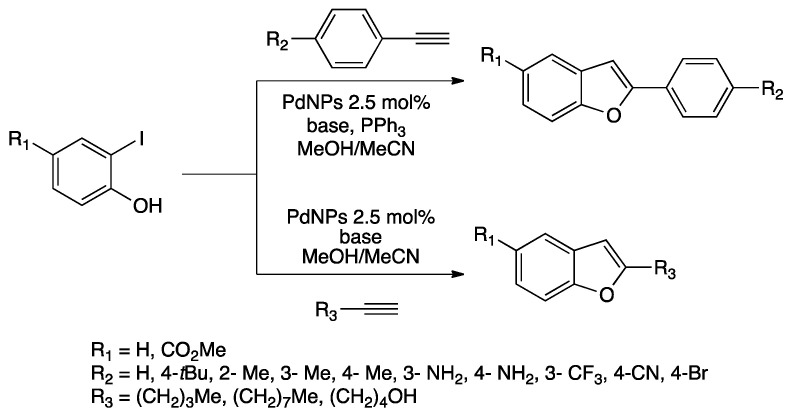
Synthesis of substituted benzofurans catalyzed by PdNPs in MeCN/MeOH [145].

Preparation of substituted benzofurans is a current topic in academic and industrial research in particular for targeting compounds with potential biological properties [141,142]. Chand and co-workers prepared PdNPs by reduction of Pd(OAc)_2_ in a mixture of MeCN/MeOH without adding any stabilizer. The as-prepared nanomaterial was characterized by (HR)-TEM and PXRD showing nanoparticles and some agglomerates of crystalline Pd(0) [143]. Considering that the as-prepared catalyst was efficient for Sonogashira and Suzuki–Miyaura couplings, the authors investigated the tandem Sonogashira/cyclization reaction to afford substituted benzofurans (Scheme 33) [143,144,145]. Triphenylphosphine was used as co-ligand in the case of aryl-substituted benzofurans. Employing the PdNPs dispersed in MeCN/MeOH, a large variety of benzofuran derivatives was obtained (16 examples, 68–88% yields). In addition, symmetrical dibenzofurans were obtained from 1,3-diethynylbenzene. Tuning the reaction conditions, monobenzofuran was obtained, permitting to prepare unsymmetrical dibenzofurans or derivatization through Sonogashira coupling with sterically hindered aryl halides. Moreover, substituted benzofuran containing bromide was further functionalized by Suzuki–Miyaura coupling with the same catalyst. PdNPs in MeCN/MeOH were recycled without any sign of deactivation for four runs.

Starting from 2-iodobenzyl alcohol instead of 2-iodophenol enables the synthesis of dihydroisobenzofurans via Sonogashira cross-coupling/cyclization tandem reaction [146]. Using the same catalytic system, Chand and co-workers prepared a large number of examples obtaining good yields under mild conditions (2 mol% Pd, MeOH/MeCN, 60 °C). The synthesis of dihydroisobenfurans was coupled to Suzuki–Miyaura or Hiyama cross-couplings to prepare further functionalized heterocycles.

**Scheme 34:**
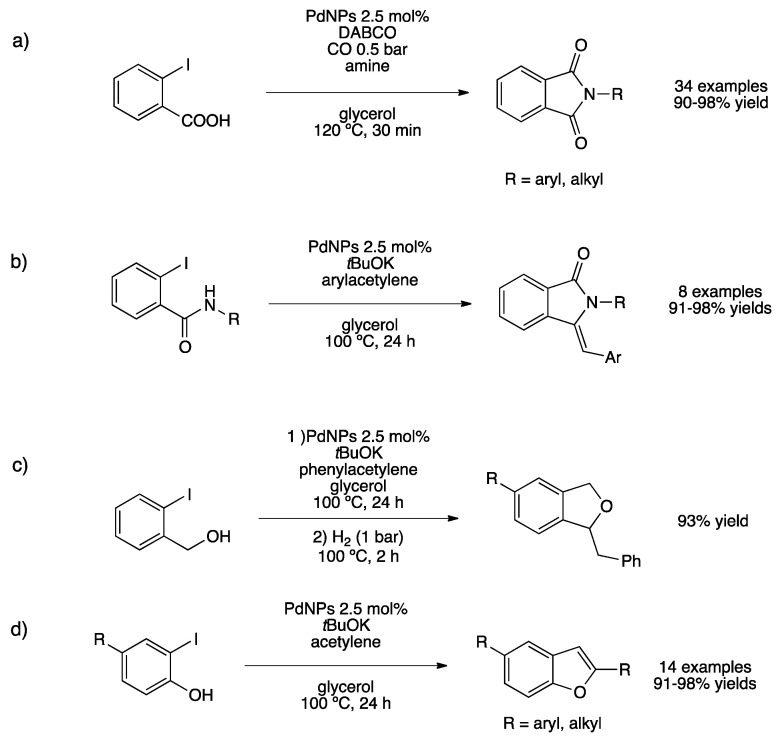
Synthesis of heterocycles catalyzed by pre-formed PdNPs stabilized by TPPTS in glycerol (**a**,**b**,**c**,**d**) [147].

We investigated the synthesis of different heterocycles by one-pot tandem processes catalyzed by PdNPs immobilized in glycerol [147]. The catalyst described above (PdNPs/TPPTS) led to the synthesis of a plethora of nitrogen and oxygen-containing heterocycles. Phthalimides were prepared from 2-iodobenzoic acid and both aryl and alkyl amines under low carbon monoxide pressure (Scheme 34a). Under the same conditions but in the absence of CO, isoindolinones and isoquinolindiones were prepared in excellent isolated yields. *Z*-(aryl)isoindolinones were prepared through Sonogashira coupling/cyclization tandem reaction affording the desired products in quantitative yields (Scheme 34b). The same procedure was employed to prepare dihydroisobenzofurans starting from 2-iodobenzylalcohol in a sequential manner under hydrogen pressure and aryl- or alkyl-substituted benzofurans starting from 2-iodophenol (Scheme 34c,d). The catalytic glycerol phase was recycled up to 10 runs without loss of catalytic behavior.

This synthetic strategy also provides bis(heterocyclic) phthalimide-benzofuran by combining the carbonylative coupling and Sonogashira/cyclization reaction. Moreover, the same one-pot procedure followed by hydrogenation reaction afforded saturated isoindolinones and isobenzofurans in high yields.

**Scheme 35:**
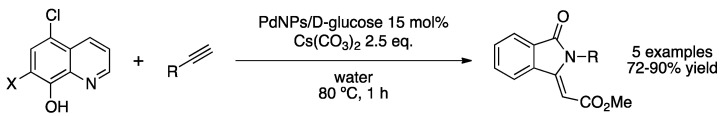
Synthesis of isoindolinones from quinoline derivatives catalyzed by PdNPs stabilized by D-glucose in water [148].

Sen and co-workers prepared PdNPs in water stabilized by D-glucose. Reduction of H_2_[PdCl_4_] with hydroxylamine provided crystalline spherical nanoparticles with an average size of 6 nm [148]. PXRD showed the *fcc* structure of Pd(0) with no signs of crystalline palladium oxides. The aqueous colloidal solution was used for a one-pot Sonogashira/cyclization reaction. Starting from different benzylamides, for short reaction times and using a high metal load (15 mol% Pd), the desired isoquinolinones were obtained in high yields. The same catalytic system provides substituted benzofurans from a quinoline ring bearing an alcohol and a halogen in relative *ortho* positions (Scheme 35). The catalytic aqueous phase was recycled up to five runs; after the sixth run, the yield dropped to 15%, probably due to the agglomeration of nanoparticles.

**Scheme 36:**
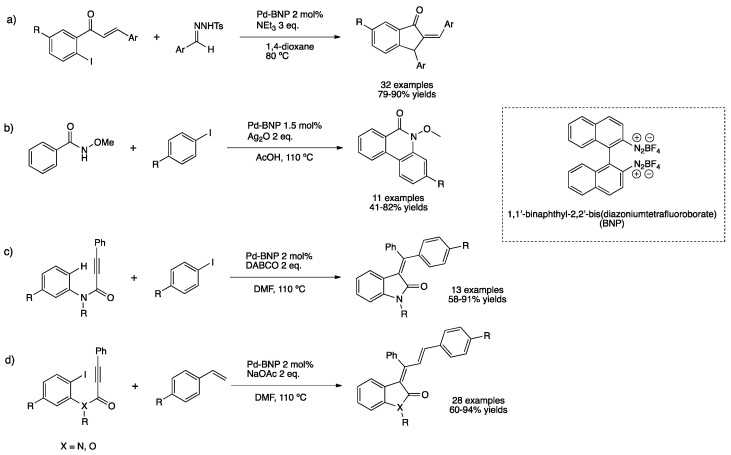
Synthesis of heterocycles catalyzed by PdNPs stabilized by BNP (**a**–**d**) [152,153,154,155].

Sekar et al. prepared PdNPs capped with 1,1′-binaphthyl-2,2′-bis(diazoniumtetrafluoroborate) ligand by reducing H_2_[PdCl_4_] with sodium borohydride in toluene [149]. The as-prepared nanoparticles were characterized by TEM, showing well-dispersed small particles (3.3 ± 0.9 nm); this nano-catalyst exhibited high activity for the direct annulation of benzamides, aminocarbonylations and cross-coupling reactions [149,150,151]. PdNPs stabilized by binaphthyl ligand were applied as well for the synthesis of indanones through one-pot carbene migration/Heck type cyclization reaction (Scheme 36a) [152]. Indanones can be further functionalized into more useful heterocycles via classical organic transformations. The catalyst was recycled five times without any sign of agglomeration, as shown by TEM analyses after the reaction. The same catalyst in the presence of silver oxide permitted the synthesis of phenanthrinidones from benzamides and aryl halides, obtaining high yields (Scheme 36b) [153]. Employing the same catalyst, single-step stereoselective synthesis of allylideneoxindoles and allylidenebenzofuranes was achieved as well as a stereoselective domino synthesis of oxindoles from anilides (Scheme 36c,d) [154,155].

#### 2.2.4. Miscellaneous

**Scheme 37:**
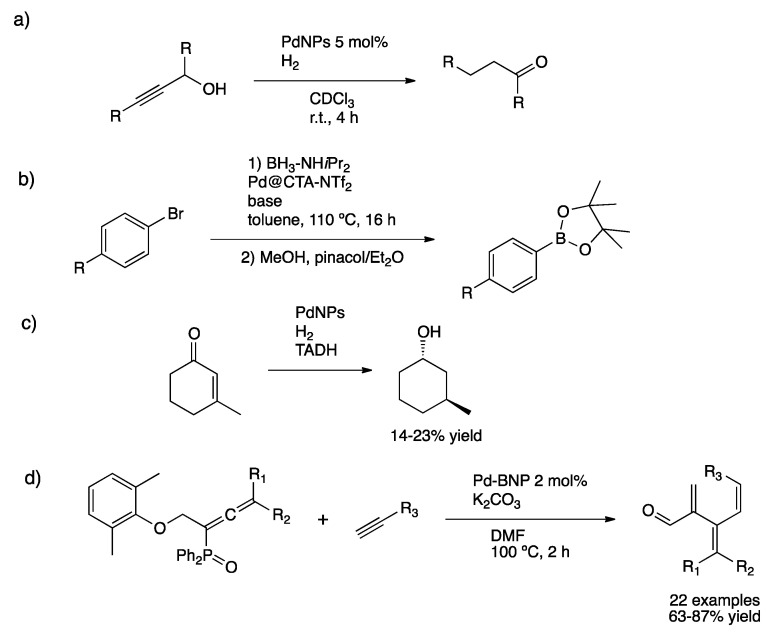
Tandem isomerization/hydrogenation of propargyl alcohols (**a**) [156]. One-pot borylation reaction using borane as boron source (**b**) [158]. C=C/C=O reduction catalyzed by a dual Pd–TADH catalytic system (**c**) [159]. Alkynylation/reduction from allenylphosphines and terminal alkynes over Pd–BNP (**d**) [161].

Shon and co-workers studied the semi-hydrogenation/isomerization of propargyl alcohols as an efficient methodology to prepare ketones (Scheme 37a) [156]. PdNPs capped with dodecylthiosulfate ligands were prepared, exhibiting a lower surface coverage than those prepared with dodecanethiol and thus a higher number of available active sites [157]. Actually, characterization enabled the proposal of an idealized formula Pd_1289_(SC_12_H_25_)_164_ with an average size of 3.4 nm and a moderate ligand coverage (ca. 0.34 ligand/Pd surface atom). Reaction occurred via isomerization of propargyl alcohol to the corresponding allyl alcohol and then selective hydrogenation of the carbon–carbon double bond.

Pucheault and co-workers investigated the dehydrogenation of diisopropylamine-borane followed by arylation into a haloarene and esterification with pinacol, leading to the formation of boronates catalyzed by PdNPs stabilized by cetyltrimethylammonium bis(trifluoromethane)sulfonimide (CTA-NTf_2_) (Scheme 37b) [158]. Although moderate yields were obtained, the use of borane adduct seems a promising way for borylation purposes. D’Alessadro and co-workers examined the palladium-catalyzed reduction of C–C double bond followed by carbonyl reduction catalyzed by thermostable alcohol dehydrogenase [159]. This dual catalytic system proved to be active; however, yields were low towards the asymmetric alcohol (Scheme 37c). Wu et al. prepared PdNPs using a modified-Sekar synthetic methodology obtaining smaller nanoparticles (ca. 2.5 nm), which were applied in alkynylation/reduction tandem catalysis [160,161]. Under hydrogen pressure and using a low metal load (2 mol% Pd), a variety of products were isolated from allenylphosphine oxides and terminal alkynes (Scheme 37d).

## 3. Copper Nanoparticles

### 3.1. CuNPs Immobilized on Solids

#### 3.1.1. Multi-Step Redox Reactions

**Scheme 38:**
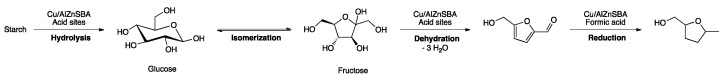
Copper-catalyzed synthesis of MFA from starch [163].

Analogously to PdNPs, CuNPs have been studied for multi-step processes involving reduction and oxidation steps in order to obtain value-added products. Luque et al. investigated the microwave-assisted synthesis of 5-methylfurfuryl alcohol (MFA) from sugars using Cu(0)NPs supported on zinc-modified aluminosilicates [162]. Thus, glucose was completely transformed, in less than 10 min into 5-methylfurfuryl alcohol (>70% selectivity) through formic acid dehydration followed by Cu-catalyzed selective hydrogenation of 5-hydroxymethylfurfural. Incorporation of Zn into Al-SBA structure reduced the acidity of the catalytic material and tuned the selectivity even at very low copper contents (0.2 wt%). They employed the same catalytic system for the synthesis of MFA starting from starch, which underwent hydrolysis to give glucose, showing the robustness of the material (Scheme 38) [163]. In addition, this catalytic system was reused five times, preserving the catalytic behavior.

Luque’s group also reported the synthesis of vanillin from trans-ferulic acid using CuNPs supported on titania as the catalyst and H_2_O_2_ as the oxidant [164]. CuNPs/TiO_2_ (0.3% Cu) was prepared by solvothermal methodology in ethanol in the presence of nitric acid. The as-prepared material exhibited an excellent activity at short times and high selectivity towards vanillin (61%) under acetonitrile reflux. Wang et al. were interested in the selective hydrodeoxygenation of vanillin, an important aromatic monomer of lignin (see Section 2.1.3), to afford 2-methoxy-4-methylphenol [165]. Highly dispersed and spherical narrow-sized CuNPs (31.2 ± 3.2 nm) prepared by carbothermal reduction and supported on activated carbon, were employed in this transformation, achieving complete conversion and 93.2% selectivity towards the desired product (reaction conditions: 120 °C, 2 bar H_2_, 5 h, 15 wt% Cu). Moreover, 2-propanol was used as a hydrogen donor obtaining similar results under slightly harsher conditions (180 °C, 5 h). Importantly, both activity and selectivity of the catalyst remained stable after five catalytic runs.

One-pot reductive-acetylation of nitroarenes in water catalyzed by magnetic copper hydroxide nanoparticles was reported by Hosseini and co-workers [166]. CuCl_2_ precursor was impregnated on Fe_3_O_4_ nanoparticles under an extremely basic solution to afford Cu(OH)_x_@Fe_3_O_4_ nanoparticles, as shown by PXRD analysis. The reductive acetylation of nitroarenes in the presence of sodium borohydride and acetic anhydride was conducted under very mild conditions (0.06 mol% Cu, 60 °C, 6–20 min) to afford *N*-arylacetamides in excellent yields (89–94% for 14 examples). Moreover, the catalyst was recovered with a simple magnet and reused for nine runs without significant loss of activity.

**Scheme 39:**
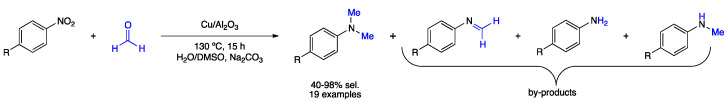
Cu/Al_2_O_3_ catalyzed selective *N*-methylation of nitroarenes with *p*-formaldehyde [169].

*N*-methylation is a powerful synthetic approach for late-stage functionalization, permitting the modulating of the biological and pharmaceutical properties of molecules; however, traditional procedures operate with powerful but carcinogenic methylating agents, such as alkylnitrosamides [167,168]. Yang and co-workers addressed this issue using *p*-formaldehyde and CuNPs dispersed on amorphous Al_2_O_3_ [169]. CuAl-layer double hydroxide was synthesized by co-precipitation prior to being reduced under H_2_ at high temperature. The resulting material was composed of a mixture of metallic Cu and Cu_2_O nanoparticles (ca. 20 nm), highly dispersed on alumina. Cu/Al_2_O_3_ catalyzed the selective *N*-methylation of a variety of nitroarenes via reduction of the nitro group, condensation of amine and *p*-formaldehyde, reduction of imine, condensation of a second molecule of *p*-formaldehyde and reduction to afford the desired product (Scheme 39). The authors proved the synergistic effect of Cu(0) and Cu(I) species in order to enable the different reaction steps. Actually, Cu(0) or Cu(I) as independent catalysis resulted in decreased activity. Slight deactivation of the material was observed after 10 runs while preserving the selectivity.

**Scheme 40:**
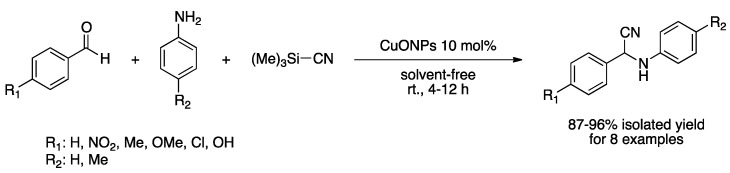
CuONPs catalyzed Strecker synthesis of α-aminonitriles [170].

Saha et al. prepared CuONPs by thermal decomposition of a previously synthesized two-dimension copper organic framework (2D-(Cu)COF) [170]. 2D coordination polymer was characterized by single-crystal XRD prior decomposition. The as-prepared CuONPs were characterized by PXRD, indicating the unique presence of CuO crystalline phase, and by HR-TEM exhibiting well-dispersed spherical nanoparticles with a mean diameter ca. 29.8 nm. This material was applied in the three-component Strecker synthesis of α-aminonitriles via condensation of benzaldehyde, aniline and trimethylsilyl cyanide under solvent-free conditions (Scheme 40). CuONPs led to excellent isolated yields of α-aminonitriles (87–96%) in very short times working at 10 mol% Cu. Reusability of the catalyst was demonstrated since no loss of activity was detected after 10 runs.

Selective conversion of coconut oil to fatty alcohols catalyzed by Cu/SiO_2_ was reported by Zhao and co-workers [171]. CuNPs preparation method was critical to obtain an active catalyst. While impregnation and deposition–precipitation methods afforded inactivated materials, hydrothermal synthesis enabled the preparation of spherical CuNPs (6.1 nm) suitable for the catalytic transformation of methyl laureate into lauryl alcohol. Actually, the authors demonstrated that it is necessary to combine Cu(0) and Cu_2_O nanoparticles in order to obtain maximum catalyst efficiency.

#### 3.1.2. Synthesis of Heterocycles

**Scheme 41:**
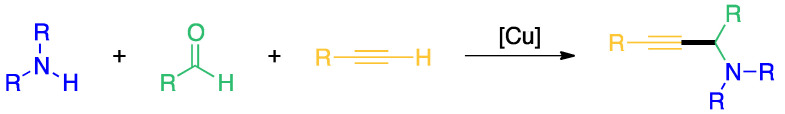
General synthesis of propargylamines via A^3^-coupling catalyzed by Cu-based catalysts.

As explained above (see Section 2.1.4 and Section 2.2.3), synthetic protocols to afford heterocycles are of great importance in organic chemistry due to their natural occurrence and biological properties. Propargylamines are frequent skeletons in nature and play key roles as intermediates in the preparation of nitrogen-based biologically active compounds such as peptides, β-lactams, drugs and oxotremorine analogs [172]. In order to prepare propargylamines, the three-component coupling of aldehydes, alkynes and amines (A^3^-coupling) represents a convenient protocol (Scheme 41) [173]. Doroodmand et al. addressed the A^3^-coupling using CuNPs supported on carbon as an effective heterogeneous catalyst in water [174]. Impregnation of CuI on activated carbon resulted in a nanostructured mixture of Cu(0) and Cu(I) species. The as-prepared material exhibited high activity at room temperature for a variety of amines and alkynes in their coupling with formaldehyde (78–91% isolated yields for 21 examples), and the protocol was extended to the coupling of aliphatic aldehydes.

Li et al. prepared Cu(0) incorporated on magnetic hydroxyapatite nanoparticles applied to A^3^-coupling of 2-aminopyridine [175]. Full characterization of the material showed the presence of 40 nm cubic Cu(0) nanoparticles, well-dispersed throughout the support. This catalytic material exhibited high activity, allowing the synthesis of a variety of imidazole[1,2-*a*]pyridines.

**Scheme 42:**
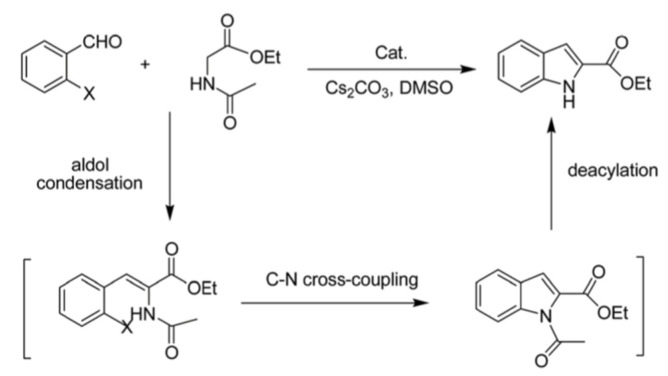
Cu-catalyzed cascade sequence for the synthesis of indole-2-carboxylic esters [178].

Indole moieties are also found in several synthetic alkaloids; thus, the development of new low-cost synthetic strategies is meaningful [176]. In particular, indol-2-carboxylic esters found applications as antimicrobial and anticancer drugs [177]. Contrary to homogeneous Cu-based catalysts generally used in the synthesis of indole-2-carboxylic esters, CuNPs can facilitate the metal separation from organic products, particularly important for biological applications. Zhou et al. synthesized CuNPs on ammonium-functionalized montmorillonite (AF-MMT) [178]. AF-MMT retained its layered structure after metal loading and could be easily dispersed in polar solvents. TEM showed that Cu loading on support resulted in slightly agglomerated lamellar particles constituted of both Cu(II) and Cu(0) species, as evidenced by XPS. Starting from different 2-chloro and 2-bromoaldehydes and ethyl acetamidoacetate, indole-2-carboxylic esters were obtained in very good yields (68–91%) under relatively smooth conditions. The catalyst was reused six times; however, TEM images showed smaller particles and leaching could be detected. The reaction involves three consecutive steps: aldol condensation, intramolecular C–N bond formation and diacylation (Scheme 42).

**Scheme 43:**
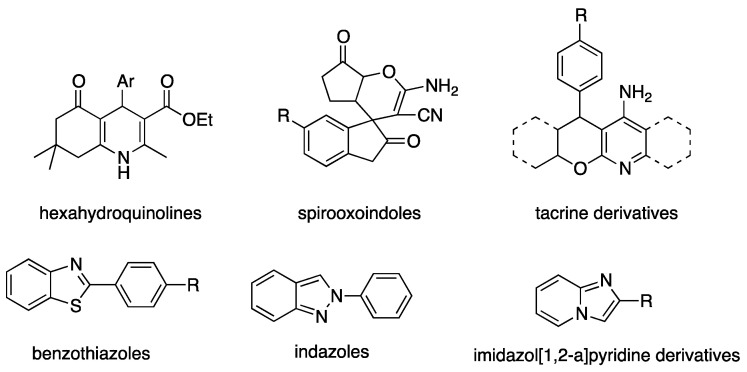
CuNPs catalyzed the synthesis of heterocycles [175,180,181,183,184,185].

Hexahydroquinolines are applied in the field of pharmaceuticals, in particular as antihypertensive drugs (Scheme 43) [179]. Pawar and co-workers prepared a variety of substituted hexahydroquinolines under ethanol reflux by reacting aldehyde, dimedone, ethyl acetoacetate and ammonium acetate, achieving high yields (82–94%) within 2 h [180]. For this purpose, they synthesized CuNPs loaded on carbon microspheres by carbonizing a styrene-based, chelate-forming cation exchange resin loaded with Cu(II) ions. The resin provides both a reductive environment suitable for the formation of metallic Cu and structured support to favor the distribution of copper ions resulting in small CuNPs. PXRD evidenced the presence of merely crystalline Cu(0), while Raman spectroscopy and SEM both exhibited resin carbonization. Pawar and co-workers employed the same catalyst for a three-component synthesis of spirooxoindole derivatives (Scheme 43) [181]. Actually, more than 20 compounds were prepared in ethanol/water reflux, achieving high yields (>86%) in less than 60 min. Tacrine derivatives are among the most investigated molecules for the treatment of Alzheimer’s disease (Scheme 43) [182]. In order to prepare tacrine derivatives avoiding intermediates isolation, Seyedmousavi and co-workers developed a Cu/MOF-catalyzed domino one-pot three-step synthesis starting from aldehyde, malonitrile, hydroxynaphthalene-1,4-dione and cycloketone [183]. CuNPs supported on MOF were highly active towards the formation of tacrine derivatives bearing different functional groups. The catalyst was reused five times with a significant decrease in activity.

Paul and co-workers prepared magnetic CuNPs coated on ethylenediamine functionalized silica (Cu(0)-Fe_3_O_4_@SiO_2_/NH_2_cel) as an efficient air-stable and water-dispersible catalyst for the synthesis of 2-substituted-benzothiazoles [184]. CuNPs were evidenced by TEM as well-dispersed spherical 9 nm particles, while PXRD showed the presence of crystalline Cu(0). The high efficiency of the prepared catalyst enabled the preparation of several target molecules in high yields. The catalyst was easily separated due to its magnetic properties and reused six times without loss of activity. A simple procedure for the synthesis of CuNPs over charcoal was employed by Khalifeh and co-workers to be applied in the synthesis of *2H-*indazole derivatives, a motif found in benzimidazoles and 2-amino-3-cyanopyridine derivatives exhibiting antidepressant, contraceptive and antiviral properties [185,186,187]. Direct copper insertion in sol-gel preparation of MCM-41 afforded a nano-sized mesoporous material, as described by Moadelli and co-workers [188]. In this case, hydroxylamine hydrochloride, aldehyde and sodium azide led to the synthesis of substituted tetrazoles in high yields (75–94%).

#### 3.1.3. Azide Alkyne Cycloadditions

**Scheme 44:**
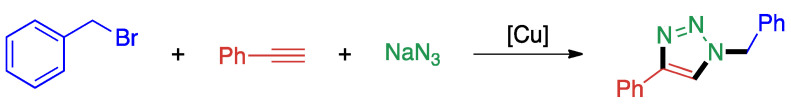
CuAAC reaction between benzyl bromide, phenylacetylene and sodium azide, often used as benchmark reaction in different reported works.

Copper-catalyzed azide–alkyne cycloadditions (CuAAC) are the most popular synthetic methods for the synthesis of 1,2,3-triazoles (Scheme 44) [189,190,191,192]. Cu(I)-catalyzed “click reaction” permits the facile preparation of 1,4-disubstituted-1,2,3-triazoles from a wide range of substrates with high selectivity, which cannot be obtained via traditional uncatalyzed methods. Concerning the application of supported CuNPs to CuAAC, Doroodmand and co-workers employed the copper over charcoal catalyst presented above, constituted of Cu(0) and Cu(I) active sites [193]. By this means, a broad scope of the desired 1,4-disubstituted-1,2,3-triazoles products in high yields was obtained. The robustness of the material allowed the reuse of the catalyst for at least 10 times. Coreño et al. deposited Cu_2_O on charcoal, obtaining agglomerates constituted of platelets; each platelet was formed by Cu_2_O nanocrystals as shown by TEM analyses [194]. Cu(I) nanoparticles over charcoal were engaged as a catalyst in the click reaction starting from benzylazides and a broad scope of alkynes affording substituted triazoles in high yields. In situ reduced graphene oxide Cu_2_O nanocomposite was prepared by Chattopadhyay and coworkers [195]. Actually, lactulose was used as both a Cu-based nanoparticle stabilizer and reducing agent of graphite oxide and copper precursor (CuSO_4_) under alkaline conditions. The surface charges of the material were determined by analyzing the zeta potential value, evidencing the presence of well-dispersed small Cu_2_O NPs(5 nm) and the interaction between Cu_2_O and support. The catalyst was applied to CuAAC reaction in water affording the desired products at 55 °C, yet a very limited scope was studied starting from benzylbromide bearing electron-withdrawing and electron-donating substituents. Catalyst recycling was successful for six runs.

Besides carbon-based supports, CuNPs supported on silica-based materials were also applied in the CuAAC reaction. Mesoporous silica KIT-5 was modified with aminopropyltriethoxysilane (APTES), in which CuI was easily grafted prior to in situ formation of CuNPs [196]. An important variety of halide derivatives, including α-bromoketones, reacted efficiently to obtained the desired substituted triazoles in moderate to good yields. Very recently, Paul et al. prepared zero-valent CuNPs supported on organic-inorganic hybrid composite obtained from starch and silica and further dopped with a nitrogen-rich tetrazole by pyrolysis [197]. The catalytic material showed the presence of well-dispersed CuNPs (ca. 10 nm by TEM) and also the presence of small amounts of Cu(II) species, but the material mainly contained Cu(0) (XPS analysis). The catalyst was applied in CuAAC reaction in water at room temperature and afforded a variety of products, from aliphatic prop-2-yn-1-ol and aliphatic bromides. CuNPs supported on silica-coated maghemite nanoparticles were prepared by reduction of CuCl_2_ affording small CuNPs (3.0 ± 0.8 nm) [198]. CuNPs/Maghemite was used for the synthesis of propargylamines via A^3^-coupling under solvent-free conditions and triazoles through CuAAC reaction in water.

Maham and Nasrollahzadeh presented a green approach for the synthesis of CuNPs supported on animal bone [199]. *Cordyline fruticose* extract was used as both a stabilizer for CuNPs and a reducing agent, while chicken leg bones composed of hydroxyapatite were used as support. Under these conditions, large and broad-sized but well-dispersed CuNPs were obtained (ca. 45 nm). The as-prepared material catalyzed the CuAAC reaction under solvent-free conditions affording the desired products in good yields within 3 h. Other supports as organic frameworks from diazonium salts and polyaniline were successfully employed in the immobilization of CuNPs and applied to CuAAC reaction [200,201].

Rawat and co-workers studied the A^3^-coupling reaction starting from picolinealdehyde derivatives in order to prepare aminoindolizines and pyrroloquinolines catalyzed by CuNPs loaded on magnetic Fe_2_O_3_ nanoparticles [202]. Hydrothermal decomposition of metal precursors promoted by glucose afforded spherical homogeneous particles mainly constituted by CuO, as proven by XPS. Cu@Fe_2_O_3_ showed a remarkable catalytic activity, in particular using glycerol as a solvent, enabling the synthesis of desired products in high yields (79–90% isolated yields within 2 h for 20 examples).

### 3.2. CuNPs Dispersed in a Liquid Phase

#### 3.2.1. Synthesis of Heterocycles

**Scheme 45:**
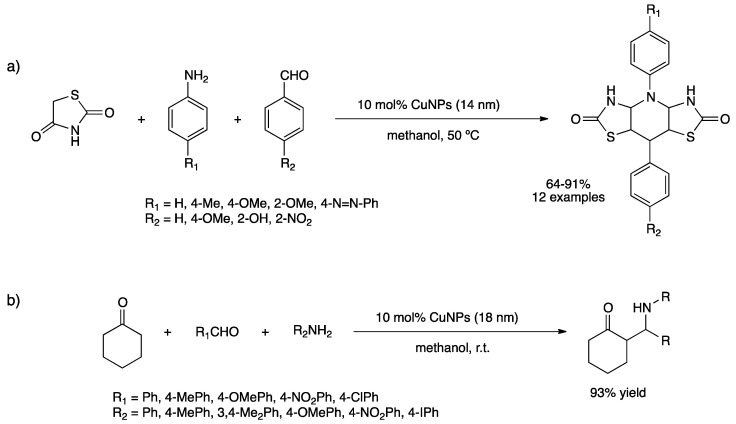
Synthesis of thiazolidine-2,4-diones (**a**) and Mannich reactions (**b**) catalyzed by reverse micellar CuNPs [203,204,205].

In 2008, Mozumdar and co-workers applied a reverse micellar droplet synthetic methodology in water for copper nanoparticles preparation, using hydrazine as a reducing agent [203]. This approach makes possible chemical reactions or co-precipitation between compounds solubilized in two different reversed micelles. Moreover, the as-synthesized MNPs were easily size-controlled by tuning the water content during the synthesis obtaining particles with a mean diameter from 10 to 80 nm as confirmed by quasi-elastic light scattering (QELS) and TEM analyses. Relatively small CuNPs (14 ± 2 nm) were applied in a one-pot three-component synthesis of thiazolidine-2,4-dione derivatives, a family of chemotherapeutic compounds that increase sensitivity to insulin and are frequently used for type 2 diabetes mellitus treatment (Scheme 45a) [204]. Under relatively mild conditions (methanol at room temperature), using 10 mol% Cu, the desired products were obtained in good to excellent yields. The same catalytic system was employed for the Mannich reaction, a three-component coupling involving a ketone, an aldehyde and an amine (Scheme 45b) [205]. Optimization study showed that, in this latter case, ca. 20 nm CuNPs were the most active.

**Scheme 46:**
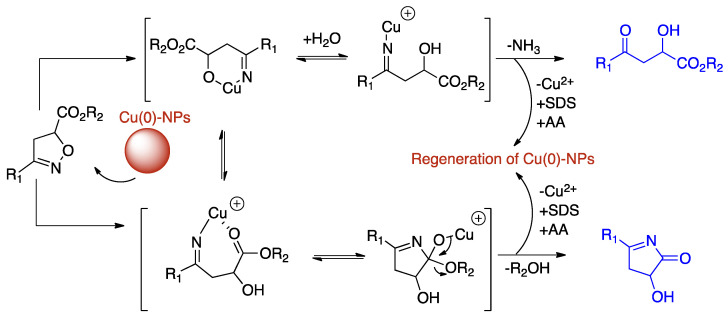
Proposed reaction pathways for the CuNPs catalyzed synthesis of β-hydroxy ketones and γ-hydroxy pyrrolinones [206]. AA stands for ascorbic acid and SDS for sodium dodecylsufonate.

Mitra and co-workers prepared CuNPs stabilized by sodium dodecylsulfonate (SDS) surfactant and reduced by ascorbic acid in the aqueous phase [206]. This methodology afforded very well-dispersed small spherical CuNPs (2–7 nm). PXRD evidenced the mere presence of crystalline Cu(0) species. The as-prepared CuNPs were effective to chemoselectively reduce ∆^2^-isoxazoline derivatives bearing an ester functionality, to afford β-hydroxy ketones. In this process, water plays a key role as an electron carrier. Increasing the reaction temperature favors the Cu catalyzed ring-opening and domino cyclization reaction to afford γ-hydroxy pyrrolinones (Scheme 46).

Juneja and co-workers prepared CuNPs stabilized by diethylenetriamine by reduction of copper salts using hydrazine hydrate as a reducing agent [207]. Under solvent-free conditions, 40 nm Cu(0)NPs afforded chromenes derivatives (10 examples, 89–94% isolated yields) via three-component coupling of aldehyde, malonitrile and 4-hydroxychroman-2-one. Later, the same group proposed a similar methodology for the synthesis of CuNPs but using PVP as a stabilizer [208]. This catalytic material was applied in the synthesis of 2,4,5-trisubstituted imidazoles affording the desired products in good yields using 10 mol% Cu. A similar methodology was applied by Zeng et al. using PEG as stabilizer and sodium hypophosphite as a reducing agent for the formation of CuNPs [209]. The alike material was used for the synthesis of imidazole[1,2-a]pyridines obtaining a large variety of products in good yields under solvent-free conditions [175].

**Scheme 47:**
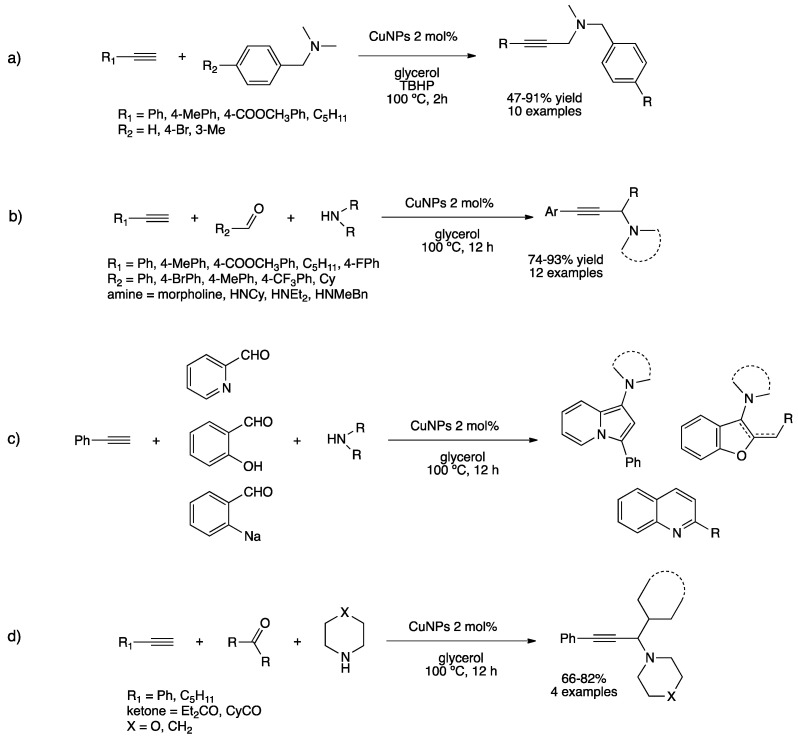
Cross-dehydrogenative couplings (**a**), A^3^-couplings ((**b**) and (**c**)) and KA^2^-couplings (**d**) catalyzed by zero-valent CuNPs in glycerol [210].

In our group, we prepared small zero-valent copper nanoparticles immobilized in glycerol and stabilized by PVP [210]. As stated above, for PdNPs, the beneficial properties of glycerol led to the synthesis of very well-dispersed nanoparticles. Reduction of [Cu(κ^2^-N,N,N′,N′-TMEDA)(µ-OH)_2_]Cl_2_ (TMEDA = tetramethylethylenediamine) under H_2_ atmosphere afforded 1.7–2.4 nm Cu(0) nanoparticles as evidenced by TEM, PXRD, UV-vis, cyclic voltammetry and XPS analyses. The as-prepared CuNPs in glycerol showed a remarkable catalytic activity for different multi-step processes, including cross-dehydrogenative couplings, A^3^- and KA^2^-couplings (ketone, aldehyde, amine) for the synthesis of propargylamines (Scheme 47). Using o-functionalized benzaldehydes in A^3^-coupling enables the synthesis of indolizines, benzofurans and quinolines. In all cases, a large scope of substrates successfully reacted, affording the desired products in high yields. Moreover, the catalyst was easily reused without any loss of activity up to five runs.

#### 3.2.2. Azide Alkyne Cycloadditions

**Figure 11:**
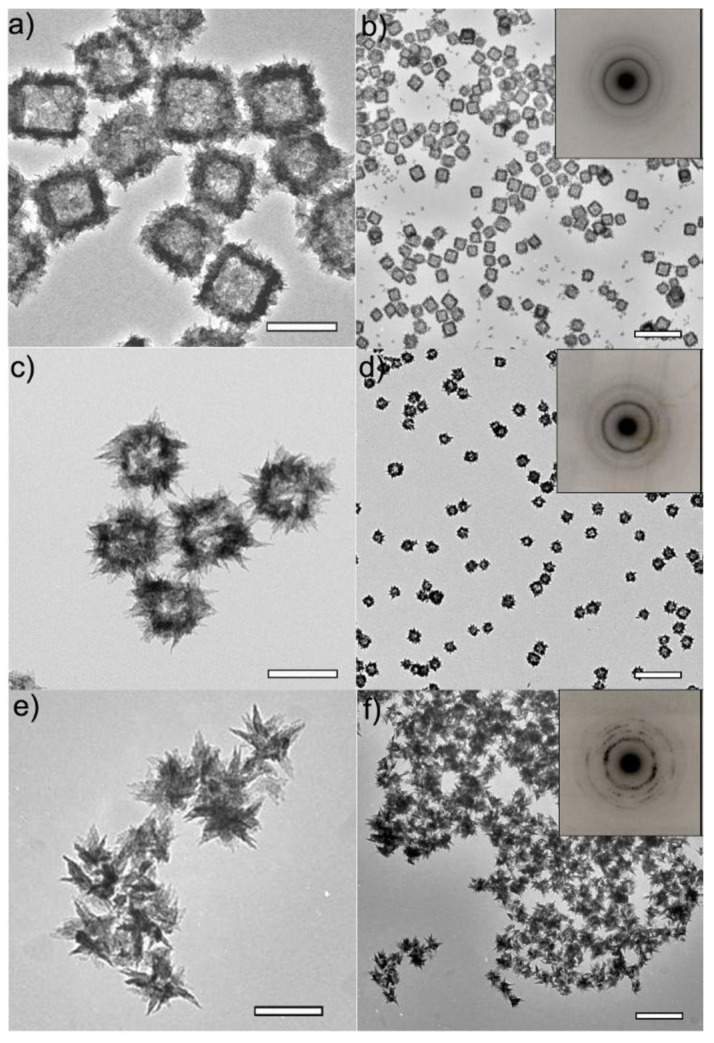
TEM images of CuO nanoparticles exhibiting the shape of hollow cubes (**a**,**b**), hollow urchins (**c**,**d**) and urchins (**e**,**f**). The insets for images (**b**), (**d**) and (**f**) show the corresponding SAED (Selected Area Electron Diffraction) patterns of single particles. Bars mean: 100 nm (**a**,**c**); 200 nm (**e**); 500 nm (**b**,**d**,**f**). Reproduced from [211] with permission from the Royal Society of Chemistry.

Unsupported CuNPs applied in azide–alkyne cycloadditions are relatively scarce. A pH-controlled synthesis of CuO hollow nanostructures prepared through the polyol process in aqueous ammonia was developed by Park and co-workers [211]. Modifying the pH permitted to obtain hollow cubes, hollow urchins and urchin-like particles (Figure 11). These materials were highly active for the CuAAC, obtaining the best results with CuO hollow nanospheres. Reaction at room temperature in water provided the desired triazoles with moderate to excellent conversions.

**Figure 12:**
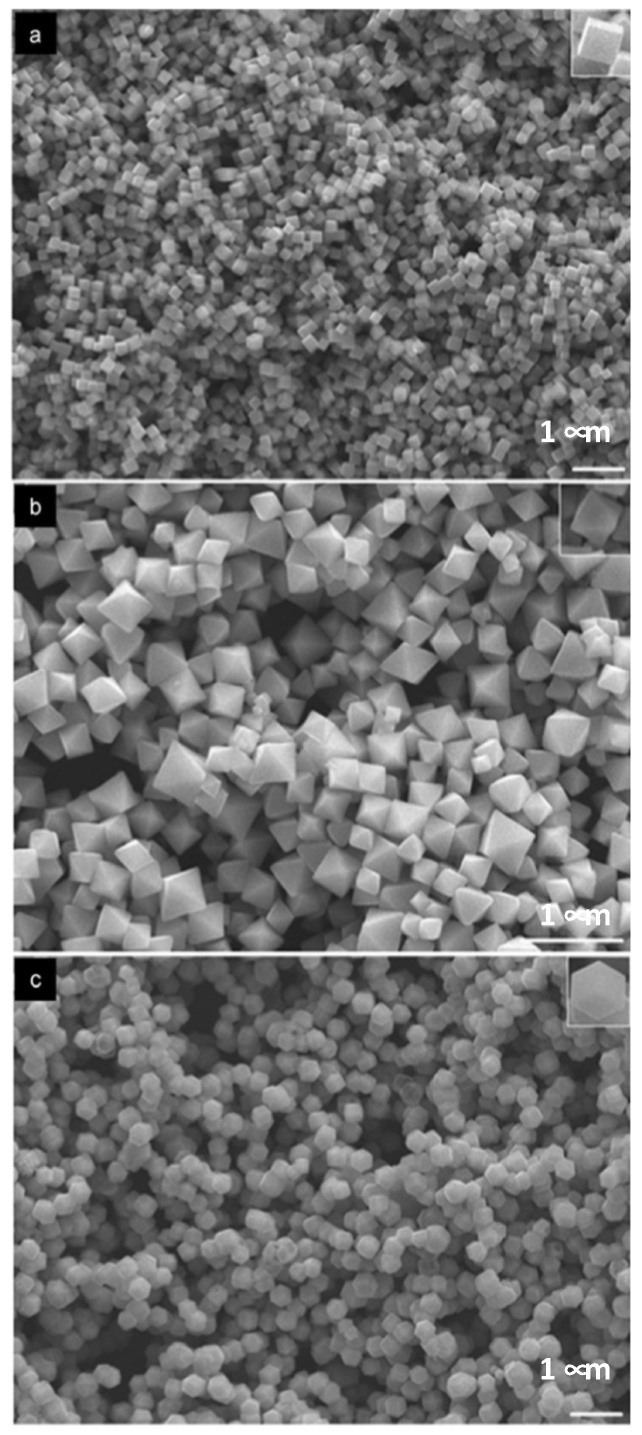
SEM images of (**a**) cubic, (**b**) octahedra and (**c**) rhombic dodecahedra Cu_2_O nanocrystals synthesized by Huang and co-workers [216]. Reproduced from [216] with permission from Wiley.

CuO nanoparticles were prepared as well through thermal decomposition of a well-defined copper complex using a thiazole picolinamide ligand [212]. The as-prepared material reacted in water to produce the corresponding triazoles with moderate to excellent isolated yields (41–95%), showing a remarkable activity compared to other supported catalytic systems [199]. Nasrollahzadeh et al. reported the synthesis of CuNPs using *Otostegia persica* leaf as both stabilizer and reducing agent, taking advantage of the presence of flavonoids and phenolic compounds present in the plant [213]. Spherical and relatively big CuNPs (45 nm) were evidenced by TEM, while PXRD showed the presence of crystalline Cu(0). CuAAC provided the desired triazoles starting from both aromatic and aliphatic halides. Very recently, Rai and Chand prepared Cu_2_O NPs using glucose from starch (*Oryza sativa*) as a reducing agent [214]. Full characterization by PXRD and XPS showed the presence of Cu_2_O species, while TEM images showed small spherical Cu_2_ONPs (9–10 nm). Under relative mild conditions (water, 100 °C, 30 min), Cu_2_ONPs catalyzed the formation of mono-triazoles bearing a free acetylene group and bis-triazoles starting from different alkynes and halides, in very good yields. Moreover, the catalytic system was be reused four times. Cu_2_ONPs stabilized with PVP were prepared by reduction of copper salt with sodium borohydride in water [215]. Well-dispersed particles (20 nm) were found together with large agglomerates (50–170 nm). Both PXRD and XPS analyses showed that nanoparticles were mainly composed of Cu(I) species. Room temperature coupling of benzyl and aliphatic azides with alkynes catalyzed by 1 mol% Cu_2_ONPs in water provided the desired products in high yields. Sharp-faced oxide nanocubes, octahedra and rhombic dodecahedra bounded exclusively by [100], [111] and [110] surfaces, respectively, were prepared by Huang and co-workers by reducing CuCl_2_ with hydroxylamine hydrochloride in the presence of SDS as stabilizer [216,217]. SEM analyses of the prepared materials showed the controlled Cu_2_O nanocrystal shapes (Figure 12). In fact, Cu_2_O rhombic dodecahedra bounded by [110] facets showed the best catalytic activity for the synthesis of triazoles, probably due to the fully exposed surface Cu atoms as demonstrated by area density analysis of the different crystal model planes. Cu_2_O rhombic dodecahedra showed high activity for preparing 1,4-disubstituted 1,2,3-triazoles with high regioselectivity, including the synthesis of rufinamide, an antiepileptic drug.

**Scheme 48:**
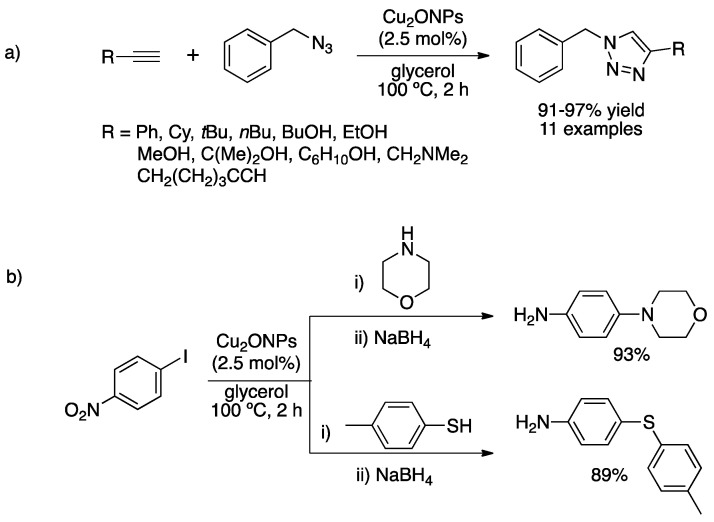
Cu_2_ONPs in glycerol catalyzed azide–alkyne cycloaddition (**a**) and coupling reactions (**b**) [110].

Xu et al. reported an efficient catalytic system based on in situ generation of Cu_2_ONPs by adding Cu(OAc)_2_ and N_2_H_4_ in the reaction media; however, they do not provide consistent evidence for the formation of nanoparticles [218]. In our group, we conducted reproducible and rigorous experiments on CuAAC reactions in order to demonstrate the key role of impurities randomly present in commercial samples of benzyl azide that triggered the formation of Cu(I) nanoparticles which are the catalytic-active species [219]. Actually, amines bearing long alkyl chains present as impurities in commercial benzyl azides stabilize the formation of Cu(I) nanoparticles in glycerol, contrary to freshly prepared benzyl azide. The in situ generated Cu(I)NPs in glycerol afforded disubstituted 1,2,3-triazoles in high yields (75–99% for 12 examples) at room temperature. Pre-formed Cu(I) oxide nanoparticles in glycerol were prepared from Cu(OAc)_2_ reduction under hydrogen pressure and using polyvinylpyrrolidone (PVP) as a stabilizer [110]. PRXD and HR-TEM evidenced the formation of cubic Cu_2_O nanoparticles without signs of Cu(II) nor Cu(0) species. The colloidal solution was active for C–N and C–S bond formation in glycerol and CuAAC reaction (Scheme 48a). Moreover, the same catalytic system was employed for sequential C–N or C–S coupling followed by nitro reduction, affording the desired products in high yields (Scheme 48b). In addition, the catalytic solution was recycled at least 10 times without loss of activity.

## 4. Palladium–Copper Bimetallic Nanoparticles

Bimetallic nanoparticles (BMNPs) consist of two metal components, exhibiting diverse types of structures, such as alloys, core–shell structures or cluster-in-cluster, among the most common arrangements [220]. The addition of a guest metal modifies the electronic properties of the active sites of the host metal by electron transfer between both partners. Furthermore, the coordination of the guest metal to the surface of the host metal gives new geometries of active sites. In some cases, the atoms of the two metals play a unique role (e.g., adsorption of different reactants or intermediates), taking advantage of the synergy between them [221,222]. BMNPs can be prepared in solution or by deposition on solid supports according to both top–down methodologies, such as sputtering ([220] and references therein), and bottom–up methodologies, such as chemical reduction, thermal decomposition of precursors, electrochemical synthesis, radiolysis or sonochemical approaches [223,224]. Concerning bottom–up methodologies, chemical reduction appears to be easily tunable by controlling the synthetic parameters which determine the final structure, and hence the properties of the obtained BMNPs. Regarding the chemical reduction synthesis, three main approaches may be considered: co-reduction of mixtures of monometallic precursors, reduction of pre-formed bimetallic complexes and sequential reduction of the corresponding monometallic precursors [220,223,224]. In fact, the synthesis way of BMNPs can result in the formation of different structures, which can trigger different catalytic behavior (structure–reactivity relationship). Therefore, the structural study of BMNPs is especially crucial for understanding both activity and selectivity of the catalytic processes. BMNPs can reveal a particular interest in catalysis mainly due to: (i) the synergy effect between both metals tuning the reactivity and (ii) the role as a multi-task catalyst for multi-step processes.

Multi-task metallic-based catalysts can be involved in tandem or sequential reactions permitting to run multiple transformations in one-pot processes, where each metal promotes a type of reactivity independently of the other metal partners; moreover, the presence of other neighboring metal sites can electronically and/or geometrically modify the properties of a given metal and in consequence, tune its catalytic properties. Thus, multi-task catalysts can be constituted of polymetallic species where each metal plays a specific role in the organic transformation [225,226,227]. On the contrary to monometallic catalysts that are limited in scope due to the high metal specificity in organic reactions [228,229,230,231,232,233,234,235], multi-task polymetallic catalysts allow combining at least two different transformations based on the specificity of each metal, and thus expand the diversity of one-pot processes [236,237,238,239]. Significantly, the synergy that can be induced between the metals modifies the catalytic reactivity in relation to the monometallic catalyst [225,226,227,236,237,238,239]. Nevertheless, to date, bimetallic catalysts have been less exploited in organic transformations in comparison with monometallic ones, mainly due to the difficulty encountered in controlling the structure of BMNPs, which strongly depends on the reaction conditions and the nature of the metal precursors.

**Scheme 49:**
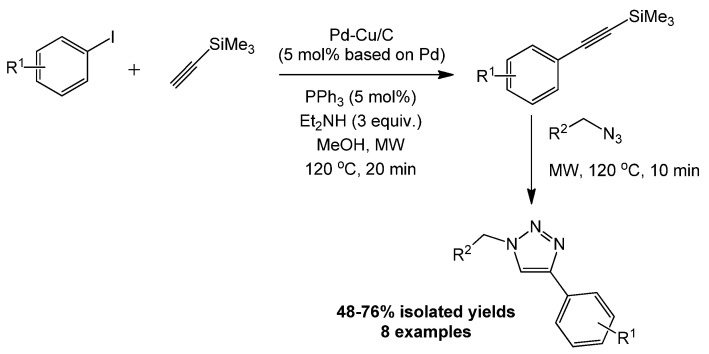
Sequential Sonogashira–AAC coupling using Pd–Cu/C as heterogeneous multi-task catalyst [244].

**Scheme 50:**
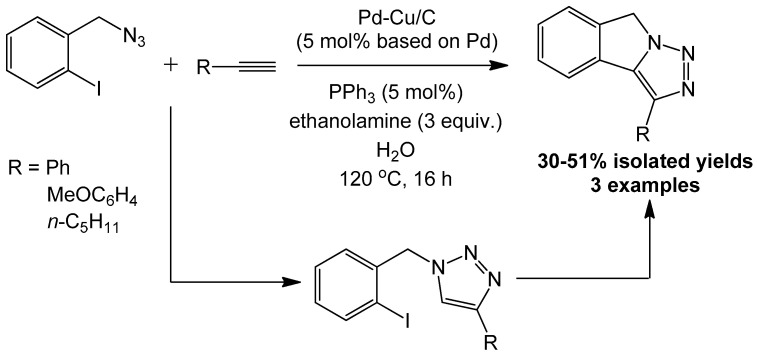
Sequential AAC–Heck coupling process using Pd–Cu/C as heterogeneous multi-task catalyst [244].

**Scheme 51:**
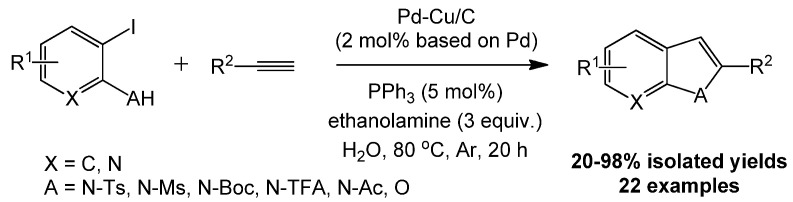
Cascade Sonogashira alkynylation-cyclization reaction to synthesize indoles, azaindoles and benzofurans catalyzed by Pd–Cu/C [245].

Among others, palladium–copper bimetallic compounds have emerged as the most efficient catalysts for multi-step reactions, mainly in C–C and C–Heteroatom bond formation catalyzed by homogeneous systems: a mixture of two metal precursors or well-defined bimetallic complexes [240,241,242,243]. Actually, bimetallic homogeneous catalysts have been applied in the synthesis of functionalized 1,2,3-triazoles from iodobenzyl azides and acetylenes, relied on Pd-catalyzed Sonogashira cross-coupling and followed by Cu-catalyzed azide–alkyne cycloaddition of the in situ generated internal alkynes. Concerning BMNPs, very few reports concern catalytic applications in multi-step reactions, probably due to the complexity of elucidating their structures. According to a similar strategy to synthesize such triazoles, Felpin et al. described PdCu-BMNPs supported on charcoal for applications in multi-step processes [244,245]. In this work, a mixture of Pd(OAc)_2_, Cu(OAc)_2_ and activated charcoal was stirred in methanol under H_2_ atmosphere (1 atm, balloon) at 25 °C for 12 h, yielding bimetallic alloys homogeneously dispersed on charcoal as observed by TEM, HRTEM and STEM-EDX (crystallite sizes in the range of 4–10 nm), with metal contents of 5.0 wt.% Pd and 3.6 wt.% Cu, as shown by ICP-MS analysis. In fact, coexistence of monometallic and bimetallic nanoparticles with 40% Pd(0) and 60% Pd(II), 30% Cu0/1+ and 70% Cu(II) was determined by XPS [244]. Such heterogeneous multi-task catalyst was applied in the synthesis of various triazoles under microwave conditions, carried out by Pd-catalyzed Sonogashira coupling followed by Cu-catalyzed azide–alkyne cycloaddition (CuAAC) (Scheme 49). More interestingly, isoindoline fused with triazoles was obtained from one-pot reactions between *o*-iodobenzyl azide and acetylenes, using PdCu–BMNPs supported on charcoal (Scheme 50) [244]. CuAAC reaction was faster than Pd catalyzed Sonogashira coupling, selectively giving the expected product from the intramolecular Heck arylation of triazoles [240,241,242,243]. Using the same heterogeneous PdCu/charcoal catalyst, heterocycles including indoles, azaindoles and benzofurans were synthesized in pure water by a cascade Sonogashira alkynylation–cyclization reaction (Scheme 51) [245].

**Scheme 52:**
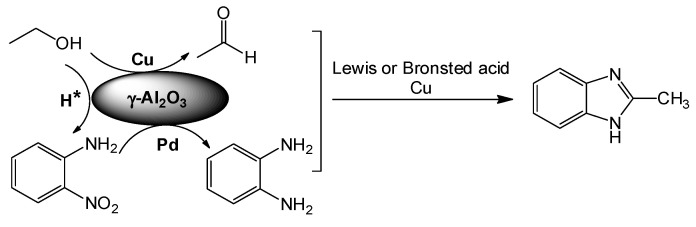
Mechanism for the direct synthesis of benzimidazole derivatives from o-nitroaniline and ethanol using multifunctional alloy Cu-Pd/γ-Al_2_O_3_ catalyst [246].

Zhang and Li reported Cu-Pd alloy/γ-Al_2_O_3_ catalyst (~5wt% Pd and ~5wt% Cu contents, with particles of a mean size of 6.9 nm) applied in hydrogen transfer reaction and sequential cyclization coupling from *o*-nitroaniline and alcohol, affording benzimidazole derivatives in high yields (up to 100% yield, 15 examples) [246]. First, Cu-catalyzed dehydrogenation of alcohol produced aldehyde and hydrogen, active species in the successive transfer hydrogenation of *o*-nitroaniline catalyzed by Pd. The resulting *o*-phenylenediamine was then condensed with aldehyde favored by the Lewis acid sites of γ-Al_2_O_3_ to give the corresponding Schiff base, and then benzimidazole compound was obtained via nucleophilic cyclization and dehydrogenation on the catalyst surface (Scheme 52). The in situ formation of *o*-phenylenediamine and aldehyde were adsorbed on the alumina surface, decreasing the catalyst acidity and thus lowering the activation energy of the reaction, resulting in faster processes and affording high yields in short times. Interestingly, Cu-Pd/γ-Al_2_O_3_ was recycled up to six times without any loss of activity (97.3% yield for the fresh catalyst and 95.8% yield after six runs in the reaction between ethanol and *o*-nitroaniline). Insignificant differences in the crystalline phase were observed between fresh and reused catalyst as proven by XRD analysis, confirming the high stability of the catalyst under multi-step one-pot conditions [246].

**Figure 13:**
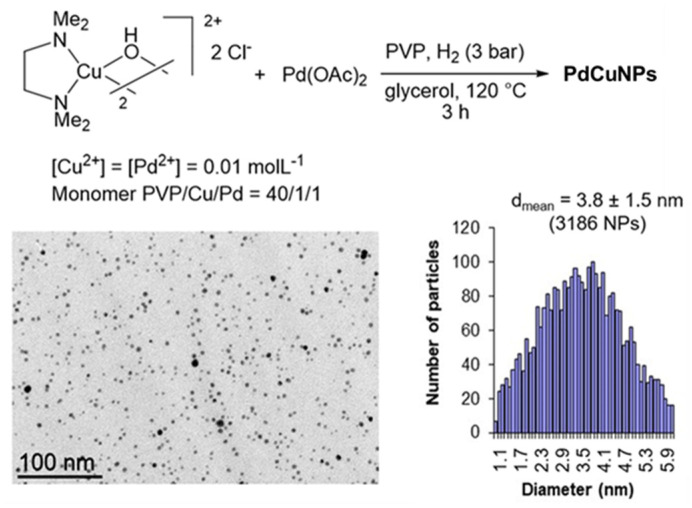
Synthesis of PdCu–BMNPs in neat glycerol by co-reduction of equimolar metal precursors, TEM micrograph and corresponding size distribution diagram of PdCu-BMNPs [247]. Reproduced from [247] with permission from the American Chemical Society.

**Scheme 53:**
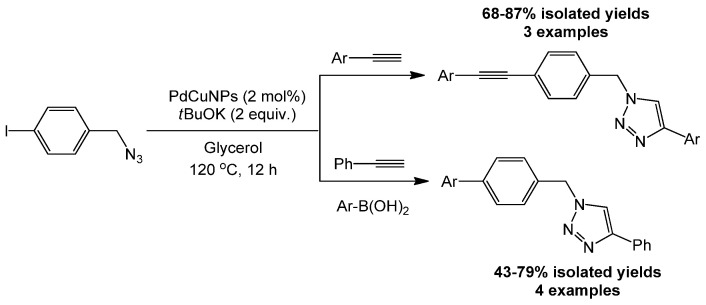
Synthesis of functionalized 1,2,3-triazoles by one-pot tandem AAC/C−C cross-coupling reactions catalyzed by PdCu-BMNPs [247].

To the best of our knowledge, our group, for the first time, reported unsupported PdCu-BMNPs acting as multi-task catalysts applied in one-pot processes [247]. In this study, well-dispersed spherical PdCu-BMNPs (ca. 3.8 nm) in neat glycerol were prepared by co-reduction of equimolar amounts of Pd(OAc)_2_ and [Cu(κ^2^-N,N,N′,N′-TMEDA)(μ-OH)]_2_Cl_2_ at 120 °C under hydrogen pressure (3 bar), using PVP as a stabilizer (Figure 13). The as-prepared BMNPs were thoroughly characterized by diverse techniques (XRD, FT-IR, XPS, HR(TEM), HAADF-STEM, EDX mapping and cyclic voltammetry), evidencing the formation of BMNPs consisting of small Pd clusters coated by copper atoms, in a cluster-in-cluster-like arrangement [247]. This catalyst exhibited enhanced activity and selectivity in semi-hydrogenation of alkynes and azide–alkyne cycloaddition in comparison with the monometallic counterparts. In particular, such BMNPs in glycerol were applied in one-pot processes combining CuAAC and Pd-catalyzed C–C cross-coupling (Sonogashira, Suzuki–Miyaura), chemoselectively affording one product from modest to high yields (Scheme 53) [247]. It was found that the reaction rate of AAC reaction was faster than that of Sonogashira or Suzuki–Miyaura cross-coupling, avoiding competitive reactions between phenylacetylene and phenylboronic acid by activation of the C-I bond of 4-iodobenzyl azide. This could be explained according to the BMNPs’ structure, which comprises multiple small Pd particles partially coated by Cu-shell. In other words, BMNPs containing Cu surface sites with enhanced electrophilic character favor the Cu-catalyzed AAC cycloaddition rather than Pd-catalyzed C–C cross-couplings, first producing triazoles and then followed by C–C cross-coupling, in agreement with the trend observed using supported Pd–Cu/charcoal catalyst (Scheme 53) [244].

## 5. Conclusions

This review systematically highlights the diverse catalytic applications of Pd- and Cu-based nanoparticles in multi-step transformations. It should be noticed that Pd-based homogeneous complexes are still of vital importance, being generally the first choice applied in catalytic organic syntheses. However, alternative catalytic systems, mostly metal-based nanoparticles, have prompted a revolutionary change in the mindset of organic chemists and also gained some promising achievements. With the exception of efficient catalytic activity of PdNPs towards hydrogenations, single steps involved in multi-step transformations have been previously well-known using homogeneous catalysts and then moved to one-pot, MNPs-catalyzed methodologies. Despite the fact of showing lower catalytic activities, metal-based nanocatalysts have recently gained attention from a sustainable facet involving recyclable ability and non-contaminated organic products with metal traces.

The reaction pathways catalyzed by MNPs have usually been modeled corresponding to the homogeneous cycles, and their mechanism remains unexplored. It has been widely admitted that metal species leached from MNPs act in a homogeneous catalytic cycle and then re-deposited on nanoparticle surface when the organic transformation is completed. In this general overview, the deep understanding of Pd-catalyzed mechanisms as well as their versatile activity displayed by the numerous contributions on PdNPs, in contrast to the less accessible CuNPs in such multi-step transformations, is shown.

Recently, organic syntheses have been gradually moving on using the first-row, transition-metal-based nanocatalysts, in particular CuNPs. Besides the low cost, copper can turn its reactivity through both one- and two-electron pathways, based on the different oxidation states [Cu(0), Cu(I), Cu(II), Cu(III)].

On the other hand, PdCu–BMNPs have been extremely appreciated as multi-task catalysts, combining at least two transformations based on the specificity of each metal. However, low success on the application of BMNPs has been reached so far, probably due to the incompatibility between the two metals and the low control on the reaction conditions. The relationship between structures of PdCu–BMNPs (alloy, core–shell structure or cluster-in-cluster) and their catalytic reactivity in one-pot processes has not been still reported, representing an exciting research area to be further developed.

In the foreseeable future, the strategy of using CuNPs and BMNPs in one-pot multi-step transformations towards target molecules will be challenging but full of opportunities to be explored.

## Figures and Tables

**Table 1:**
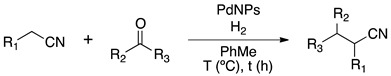
Comparison of the reported catalytic activity to the sequential condensation/reduction synthesis of α-alkylated nitriles.

	Catalyst (Particle Size in nm)	[Pd] (mol%)	P(H_2_) (bar)	T (°C)	t(h)	Ex. ^a^	Conv. (%)	Sel. (%)	Yield (%)	Ref.
1	Pd/HT (7)	1	1	80	24	14	-	-	19–99	[19]
2	Pd/SBA-15-NH_2_/ (5)	2	1	80	1	9	-	-	93–99	[20]
3	Pd/Cz-MOF-253-800 (1)	-	10	80	17	3	99	99	-	[21]
4 ^b^	Pd/C_3_N_4_-NH_2_ (3.4)	1	2	25	8	9	76–99	59–99	-	[22]
5	Pd-ZIF-8/rGO (2.8)	9	balloon	60	16	8	78–99	88–98	-	[23]
6	Pd/triazine-g-C_3_N_4_NTs (3.8)	1	balloon	65	12	8	82–99	85–99	-	[24]

^a^—Number of examples. ^b^—In EtOH.

**Table 2:**
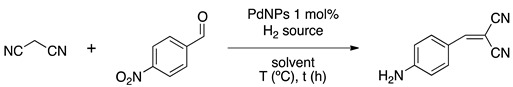
Comparison of the reported catalytic activity to the sequential condensation/nitro reduction.

	Catalyst (Particle Size)	H_2_ Source	Solvent	T (°C)	t (h)	Conv. (%)	Sel. (%)	TOF (h^−1^)	Ref.
1	Pd@IRMOF-3 (35 nm)	H_2_ 2 bar	DMF	25	24	100	86	3.6	[27]
2	HPS-C-Fe_2_O_3_-C-Pd (5 nm)	H_2_ 2 bar	DMF	80	3	99	99	n.d.	[28]
3	Pd@PDEAEMA-g-UiO-66 (8 nm)	H_2_ balloon	Toluene-water	25	0.5	95	95	9	[29]
4	Pd/DETA-APP (1–3 nm)	HCOOH	EtOH	60	5	98	95	18.7	[30]

**Table 3:**
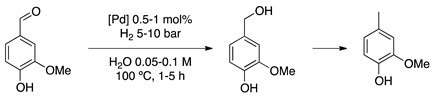
Hydrodeoxygenation of vanillin over different catalysts.

Entry	Catalyst	Size NP (nm)	TOF (h^−1^)	Ref.
1	Pd/SO_3_H-MIL-101	2.4–3.6	38.4	[67]
2	Pd@NH_2_-UiO-66	1.5–2.5	200	[68]
3	Pd/PRGO/Ce-MOF	4.0–9.6	722.3	[69]

**Table 4:**
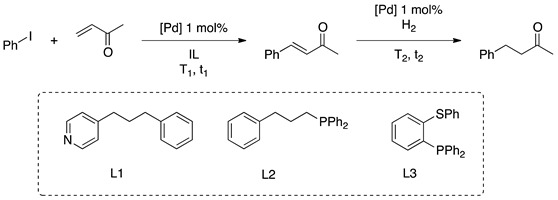
Sequential Heck coupling/reduction of 4-phenyl-2-buten-3-one in [EMI][MeHPO_3_].

Entry	Catalyst (Particle Size in nm)	T_1_ (°C)	t_1_ (h)	H_2_ (bar)	T_2_ (°C)	t_2_ (h)	Yield% ^a^	Ref.
1	Pd@[EMI][MeHPO_3_] (4)	90	3	20	90	21	50–70	[116]
2	Pd–L_1_@[EMI][MeHPO_3_]/fCNTs (4.5)	90	24	40	30	6	96	[117]
3	Pd–L_2_@[EMI][MeHPO_3_] (3)	100	20	-	-	-	77	[118]
4	Pd–L_3_@[EMI][MeHPO_3_] (3.2)	150	16	-	-	-	80	[119]

^a^—Total yield of 4-phenyl-2-butanone. EMI—1-ethyl-3-methylimidazolium; fCNTs—functionalized carbon nanotubes.
